# Defining baseline epigenetic landscapes in the rat liver

**DOI:** 10.2217/epi-2017-0029

**Published:** 2017-11-13

**Authors:** John P Thomson, Raffaele Ottaviano, Roland Buesen, Jonathan G Moggs, Michael Schwarz, Richard R Meehan

**Affiliations:** 1 MRC Human Genetics Unit, Genome Regulation, Institute of Genetics & Molecular Medicine, University of Edinburgh, Crewe Road, Edinburgh EH4 2XU, UK; 2 BASF SE, Experimental Toxicology & Ecology, 67056 Ludwigshafen, Germany; 3 Preclinical Safety, Translational Medicine, Novartis Institutes for BioMedical Research, CH-4057 Basel, Switzerland; 4 Department of Toxicology, Institute of Experimental & Clinical Pharmacology & Toxicology, University of Tübingen, 72074 Tübingen, Germany

**Keywords:** 5hmC, 5-hydroxymethylation, 5mC, CpG methylation, epigenetics, rat, strain, toxicology

## Abstract

**Aim:**

Characterization of the hepatic epigenome following exposure to chemicals and therapeutic drugs provides novel insights into toxicological and pharmacological mechanisms, however appreciation of genome-wide inter- and intra-strain baseline epigenetic variation, particularly in under-characterized species such as the rat is limited.

**Material & methods:**

To enhance the utility of epigenomic endpoints safety assessment, we map both DNA modifications (5-methyl-cytosine and 5-hydroxymethyl-cytosine) and enhancer related chromatin marks (H3K4me1 and H3K27ac) across multiple male and female rat livers for two important outbred laboratory rat strains (Sprague–Dawley and Wistar).

**Results & conclusion:**

Integration of DNA modification, enhancer chromatin marks and gene expression profiles reveals clear gender-specific chromatin states at genes which exhibit gender-specific transcription. Taken together this work provides a valuable baseline liver epigenome resource for rat strains that are commonly used in chemical and pharmaceutical safety assessment.

The liver is the main organ for the detoxification of xenobiotic compounds and as such is often a target organ for toxicity. Xenobiotic-induced hepatotoxicity is highly relevant for chemical industry but a major concern in the pharmaceutical industry where development of novel compounds can be complicated by the preclinical identification of hepatotoxic side effects. Indeed, it is estimated that in preclinical studies, about 50% of candidate compounds present hepatic effects at supratherapeutic dose [1], many of which are only detected following large-scale animal-based assays or in clinical trials. But also, in other regulated areas, it would be useful to develop novel assays for the more sensitive detection and mechanistic characterization of hepatotoxicity. Ideally, these assays should provide an improvement upon current toxicity testing regimes either by reducing the number and duration of required higher tiered animal experiments through the development of early hepatotoxicity biomarkers or by providing insight into the mechanistic basis and potential human relevance of liver toxicity.

A number of novel circulating liver injury biomarkers have recently been validated for the early detection of drug-induced liver injury (DILI) in both preclinical and clinical settings [2]. In contrast, there are no well-validated early biomarkers for delayed-onset xenobiotic-induced liver toxicities such as carcinogenicity.

Significant progress has been made in elucidating the role of genetic modifiers in preclinical and clinical drug-induced liver toxicity. This is exemplified by the identification of genetic sequence polymorphisms underlying susceptibility to acetaminophen-induced liver injury using a panel of 36 inbred mouse strains [3] and by genome-wide association studies for a diverse range of clinical DILI cases [4].

Epigenetic responses had also been implicated in hepatotoxic responses to xenobiotics [5–7]. Unlike genetic perturbations, changes to the epigenetic landscape following toxicological insult are potentially reversible. To date the potential contribution of epigenetic modifiers in susceptibility to DILI has not yet been explored but merits further investigation. One area where xenobiotic-induced liver epigenetic modifications have been extensively studied is in rodent models for drug-induced nongenotoxic hepatocarcinogenesis (reviewed in [8–12]). Importantly, these studies have elucidated epigenetic mechanisms and early biomarkers that appear within a few weeks of repeat dosing and potentially predict liver tumor formation that is only observed in the longer term (months to years).

Epigenetic profiles of methylated (5mC) and hydroxymethylated (5hmC) cytosine bases both reflect and influence the transcriptomic output of a given cell [13]. These have been utilized to investigate the well-classified mouse nongenotoxic carcinogen phenobarbital (PB) [10,14–15], and their genomic profiles have been shown to be altered in the liver following toxicological insult. Reproducible DNA modification changes were observed both over promoter regions of genes directly associated with PB metabolism, such as the CYP450 genes *Cyp2b10* and *Cyp2c55*, and at of genes with potential roles in tumorigenesis such as the Wnt signaling pathway gene *Wisp1*, the chemokine receptor *Cxcr7* and the Dlk1-Dio3 cluster of noncoding RNAs [10–11,16]. Importantly, many of the observed epigenetic changes – particularly loss of promoter 5hmC levels – were shown to occur reproducibly between two strains of mice (C57BL and C3H) following 4- or 13-week drug exposure periods and were related to the later onset of aberrant 5mC patterns in resulting long-term (35 weeks) PB-induced liver tumors [12]. Interestingly, global changes in 5hmC levels occur in the rat liver following exposure to genotoxic agents (riddelliine and aristolochic acid [17]) with reported global and promoter-specific 5mC changes occurring following exposure to nongenotoxic compounds (Limonene, dichlorobenzene and chloroform) [18]. The convenient size, physiology, genetics and behavioral characteristics of the rat have made this rodent the preferred laboratory animal in many areas of biomedical research, particularly in the field of toxicity testing. However, in order to fully exploit the potential future application of epigenetic-based research in safety assessment, it is vital that the baseline epigenetic states are fully cataloged in toxicologically relevant rat strains. We, therefore, set out to generate high-resolution datasets for genome-wide DNA modifications (5mC and 5hmC) and the histone tail modifications (H3K4me1 and H3K27ac) for both male and female rats corresponding to two commonly used outbred albino strains – Sprague–Dawley and Wistar – providing a large number of reference baseline datasets for researchers in the field. In doing so, we have highlighted the overall reproducible nature of the epigenome between rats both within and between strains. Although minor, a number of loci (both promoter and gene bodies) harbor reproducibly strong strain and gender-specific epigenetic changes – some of which occur over genes associated with metabolic functions which in turn may relate to differences in drug response between strains and genders. Finally, through the study of gender-specific gene expression differences, we highlight how combined 5hmC/H3K4me1/H3K27ac analysis may enhance the assessment of xenobiotic-induced toxicity, where strong transcriptional changes are observed. Overall, this study provides an important resource for interpreting genome-wide epigenetic-based assays in the fields of chemical and therapeutic drug safety assessment and should help to distinguish sound and relevant epigenetic changes from spontaneous and common variations.

## Materials & methods

### Rat strains

Sets of male and female Sprague–Dawley and Wistar rats (Crl:WI(Han), Charles River Laboratories, Sulzfeld, Germany) were maintained by BASF SE, Ludwigshafen Germany.

The animals were treated as usually done in a 28-day oral toxicity study following a 8- to 9-day acclimatization period. The animals were 42 ± 1 days of age at the ‘onset of the study’ and free from clinical signs. The animal facility, in which all animal work was performed, holds a certificate from the International Association for Assessment and Accreditation of Laboratory Animal Care. Rats of the same sex were housed in groups of six animals in polysulfonate cages (Tecniplast^®^, Hohenpeißenberg, Germany; floor area = ∼2065 cm2) with dust-free wooden bedding. Wooden gnawing blocks (Type NGM E-022; Abedd^®^ Lab. & Vet. Service GmbH, Vienna, Austria) were provided to the animals for environmental enrichment. The study protocols complied with the federal guidelines. Detailed clinical observations, regular health inspections, assessment of food and water consumption and the determination of the body weight were performed in weekly intervals. Hematological and clinical chemical examinations as well as urinalyses were performed toward the end of the administration period, and all standard parameters listed in OECD TG 407, paragraphs 32, 34 and 35 were evaluated for all animals. Upon completion of the maintaining period, all animals were killed by decapitation under isoflurane anesthesia after food withdrawal for at least 16 h. The exsanguinated animals were subjected to a full, detailed gross necropsy assessing and weighing all organs listed in OECD TG 407, paragraph 40. Additionally, all organs listed in OECD TG 407, paragraph 43, were preserved in neutral-buffered 10% formalin or modified Davidson’s solution for potential histopathological examination. Liver tissue of lobus sinister lateralis, spleen (one half, transversal section), parts of brain (caudal piece of cerebellum including brain stem), heart (one half, longitudinal section) and kidney, from all animals were immediately deep frozen in liquid nitrogen and stored at -80°C for further analyses at the external research facility in Edinburgh, UK.

### Methyl & hydroxymethyl DNA immunoprecipitation (MeDIP & hMeDIP)

Genomic DNA was extracted from frozen ground-up samples and fragmented to a range between 150 and 500 bp (mean 200 bp) in size using a Covaris sonicator prior to immunoprecipitation with 5hmC (Active Motif, CA, USA: Cat no. 39769) or 5mC (Eurogentec, Seraing, Belgium: Cat no. BI-MECY-1000) antibodies. For full DNA preparation and hMeDIP and MeDIP protocols, see [19]. In brief, 5mC and 5hmC-marked DNA was enriched following antibody enrichment and purified using DNA Clean and Concentrator™ (Zymo Research, CA, USA) prior to preparation for genome-wide sequencing on the Ion Proton semiconductor sequencer.

### Quantitative validation of CpG modification status

DNA modification status at a number of single CpG loci was quantified through the use of EpiMark analysis kit (New England Biolabs, MA, USA: Cat no. E3317S), following manufacturer’s instructions. 10 μg of total genomic DNA for three male and three female Wistar rats was assayed from the same extracts used for the hMe-DIP and MeDIP-seq experiments. Primers are as follows: Sult1e locus: Fw GGAGATCTATTACGGGGGAT, Rev CAATTGTGTGTAACCGTGCC; A1bg locus: Fw CAACACTCACCAGACATCTTG, Rev ACACTCACCAATGTTCCCTC; Ndrg2 locus: Fw AGTAGGAGGTGGAGTGAATG, Rev TTTTGGGGATGTTGGGGTCT (Integrated DNA Technologies, IA, USA).

### Genome-wide ChIP-seq of H3K4me1 & H3K27ac

25 μg of chromatin was isolated from Wistar rat livers and ChIP carried out using 4 μg of antibody (H3K4me1 Abcam, Cambridge, UK: Cat no. ab8895. H3K27ac Millipore, MA, USA: Cat no. 07–360), as described previously [19]. Following this, Illumina libraries were prepared by Active Motif (Active Motif, CA, USA) and samples sequenced by Illumina HiSeq prior to normalizing and processing in house.

### Analysis of gene expression datasets

We utilized a number of published microarray gene expression datasets for adult male and female Wistar rats (each n = 4) [20]. RNA was extracted from snap-frozen livers by grinding in RTL lysis buffer (Qiagen, MD, USA) prior to isolation using RNAeasy spin columns (Qiagen). Starting from 300 ng total RNA, biotin-labeled cRNA samples for hybridization on Affymetrix RaGene_2.0_ST arrays were prepared according to the protocol supplied with the GeneChip^®^ WT PLUS Reagent Kit for Affymetrix GeneChip Whole Transcript (WT) Expression Arrays (P/N 703174 Rev. 2, Affymetrix Inc, CA, USA). 3 μg fragmented ssDNA was hybridized for 16 h at 45°C. The microarrays were washed and stained with streptavidin-phycoerythrin (Molecular Probes). The fluorescent images of the GeneChips were captured with the Affymetrix GeneChip Scanner 3000. Microarray data were processed with the R/Bioconductor package ‘affy’ version 1.44.1 modified by Brainarray lab for Gene ST support. Brainarray Custom CDF ragene20st version 19.0.0 was used for summarization. Raw data were normalized using RMA as implemented in the ‘affy’ package. To determine tissue-specific and housekeeping rat gene sets (Figure 5B), we used a number of datasets from a large-scale rat RNAseq project [21] (male liver: GSM1328657, female liver: GSM1328641, male lung: GSM1328613, female lung: GSM1328598, male kidney: GSM1328581, female kidney: GSM1328565, male heart: GSM1328549 and female heart: GSM1328533) and selected the 250 top-induced genes unique to each tissue. The top 250 housekeeping genes were selected as exhibiting similarly high-expression levels in all datasets.

### Preparation & normalization of datasets

In brief, raw sequencing reads were mapped to the rat reference genome build RN6. Data were first binned across the genome into 150 bp windows and then normalized by total read count numbers. Finally, background sequencing noise was reduced through the subtraction of input (nonantibody-enriched) datasets. Datasets (Table 1) were directly compared through the levels of 5mC, 5hmC, H3K4me1 or H3K27ac over identical windows.

### Overview of bioinformatic analysis

Clustering of datasets Overall Pearson’s correlation scores were calculated between datasets. Dendrogram plots were carried out using R and distances calculated through both Euclidian and Ward methods and the resulting data (Pearson’s correlation scores) plotted as heatmaps.

#### Sliding window analysis

**Figure 1:**
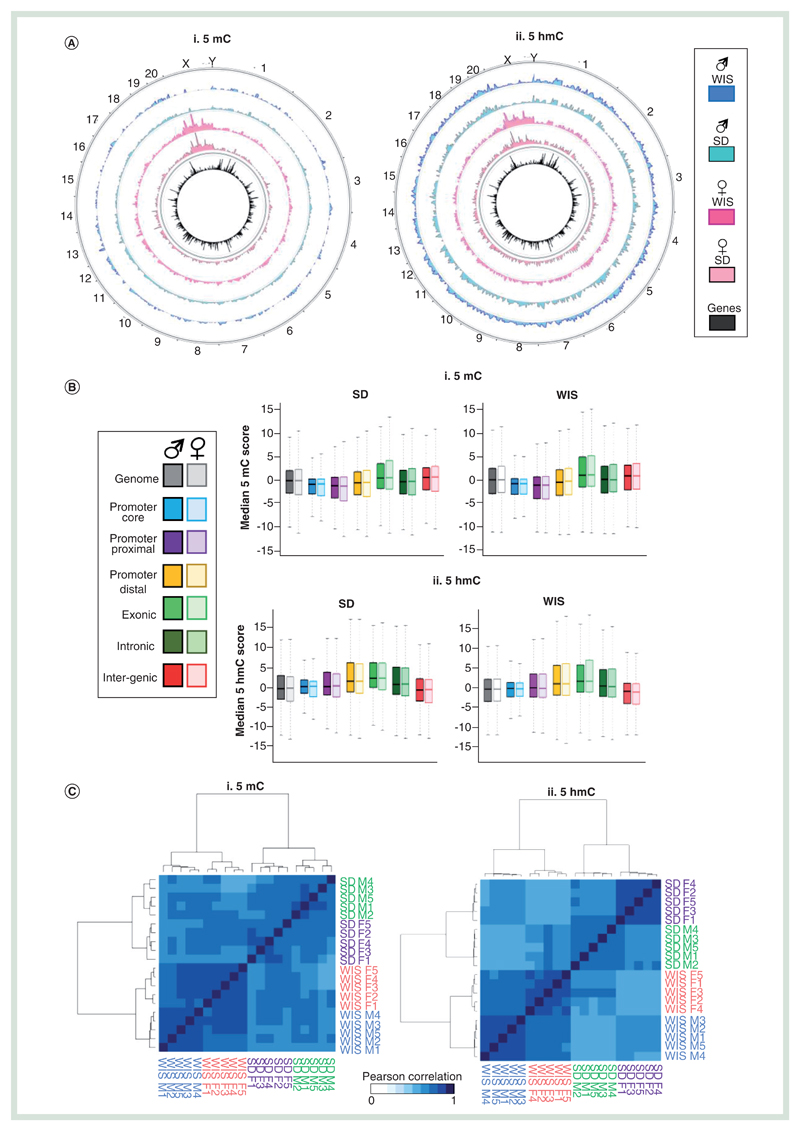

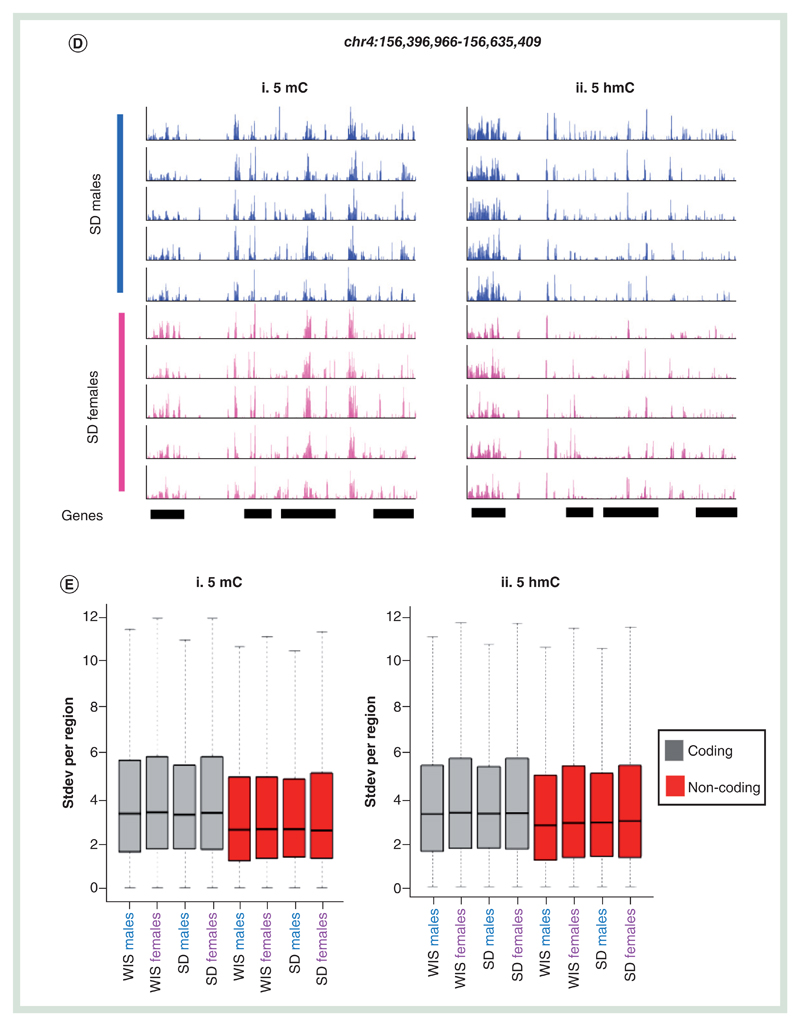

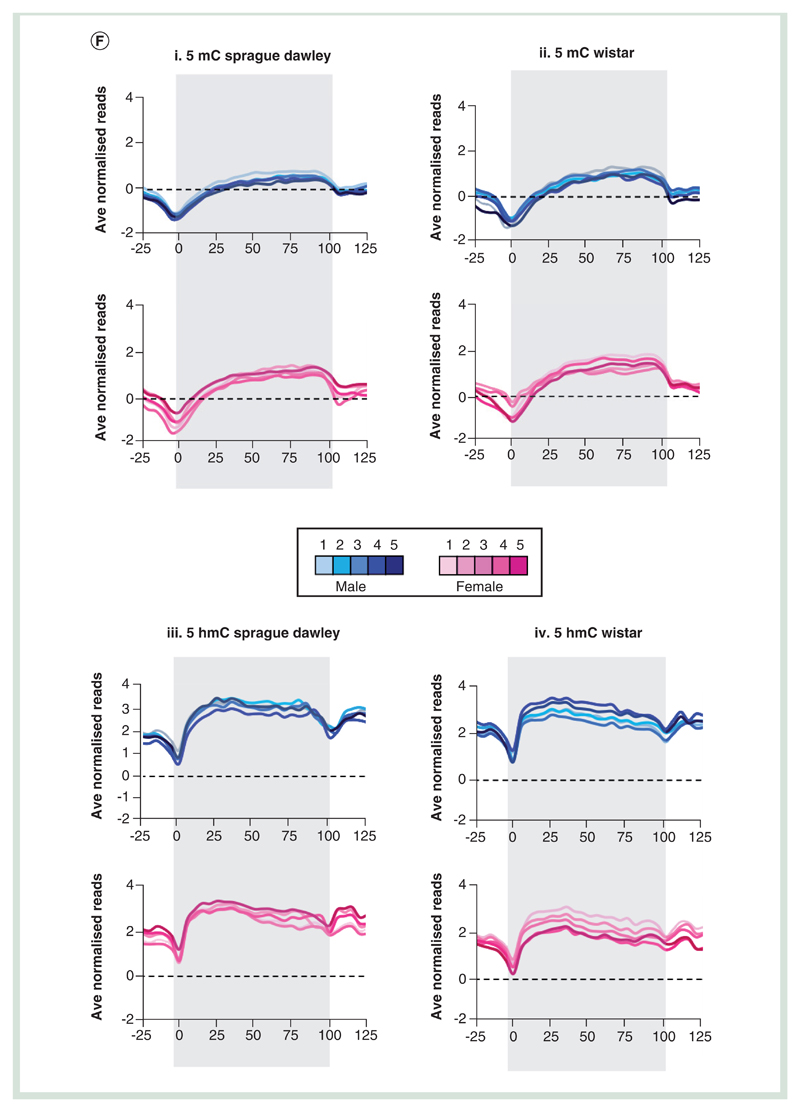
Analysis of genome-wide DNA modification patterns between Sprague–Dawley and Wistar rat livers. **(A)** Circular representation of average 5mC **(i)** and 5hmC **(ii)** datasets between male (blues) and female (pinks) Sprague–Dawley and Wistar rats. Genes are plotted in the inner circle as black bars. **(B)** Box plot for 5mC **(i)** and 5hmC **(ii)** signals across one of six genomic compartments: promoter core: TSS ± 250 bp, promoter proximal: TSS +1 kb to +250 bp, Promoter distal: TSS +2 to +1 kb, exonic, intronic or intergenic. **(C)** Pearson correlation heatmaps with hierarchical clustering for 5mC **(i)** and 5hmC **(ii)**. **(D)** Visual examples of reproducible 5mC and 5hmC patterns across multiple male (blue) and female (pink) Sprague–Dawley rat livers. Black bars represent annotated genes. **(E)** Boxplot of standard deviation across either coding (gray) or noncoding (red) regions for 5mC **(i)** and 5hmC datasets **(ii)**. **(F)** Average gene patterns for DNA modifications in each group of rat livers. **i:** 5mC Sprague–Dawley, **ii:** 5mC Wistar, **iii:** 5hmC Sprague–Dawley, **iv:** 5hmC Wistar. Plots represent average DNA modification patterns across the total gene set ± 25% gene length. Gray box indicates regions associated with the gene body. 5mC: Methylated cytosine base; 5hmC: Hydroxymethylated cytosine base; SD: Sprague–Dawley; TSS: Transcription start site; Wis: Wistar.

**Figure 2:**
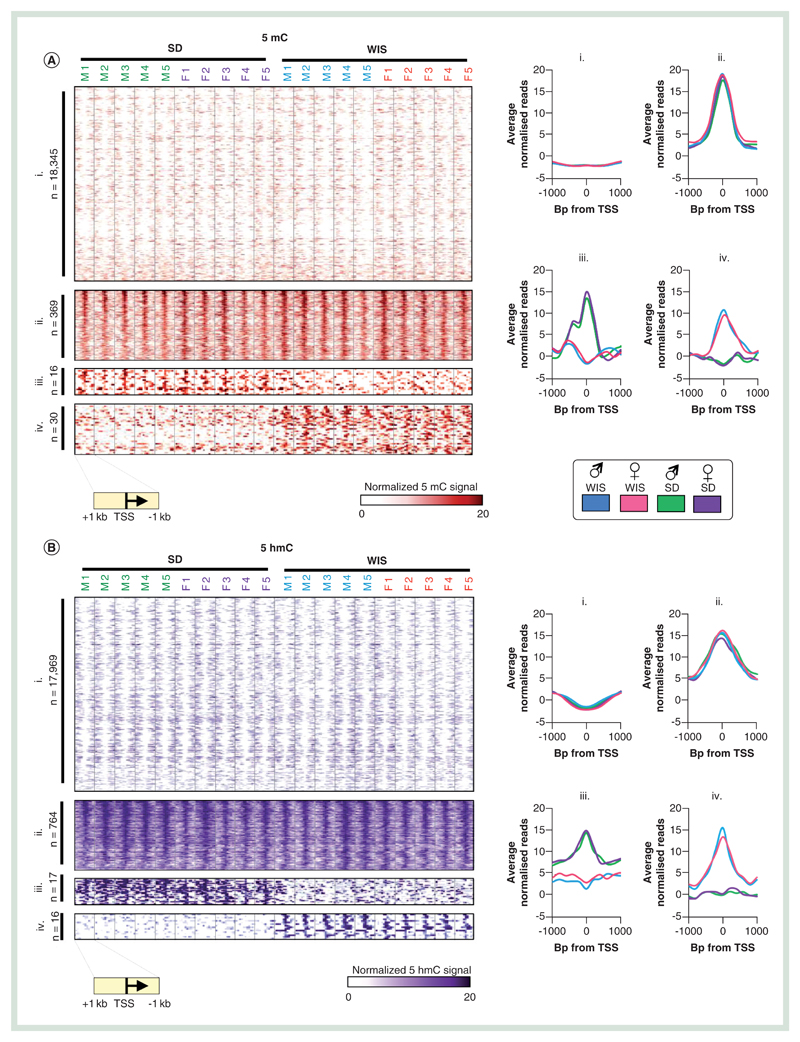

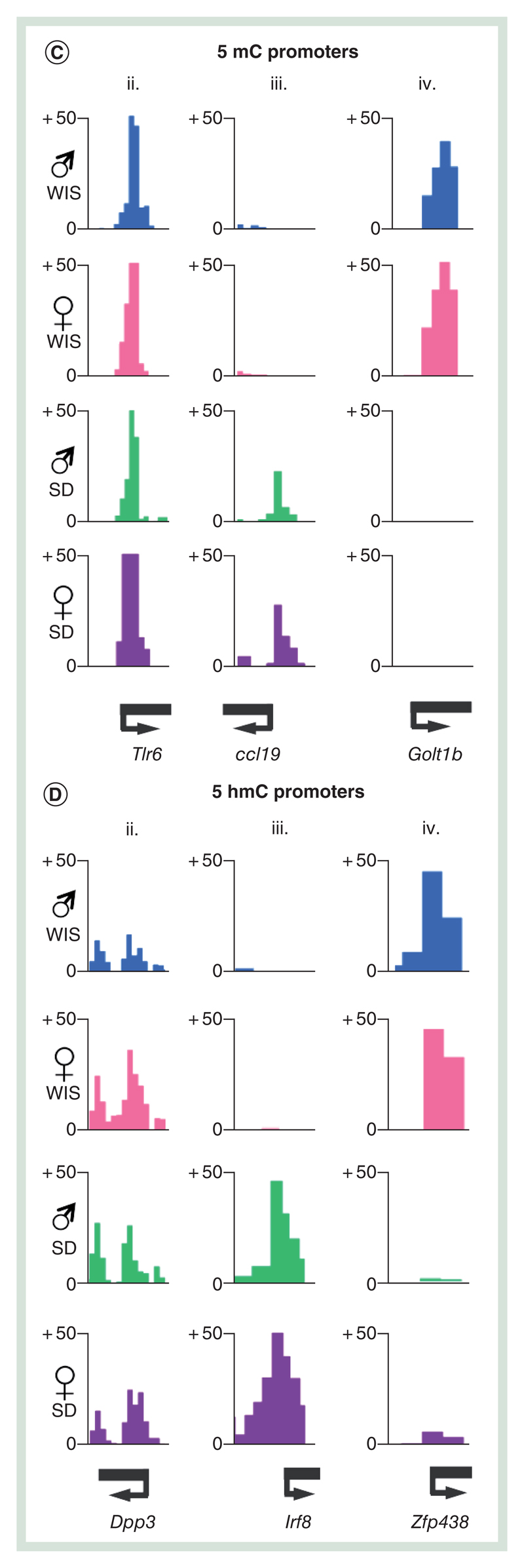
Promoter DNA modification patterns between Sprague–Dawley and Wistar rat livers. **(A & B)** Heatmap of promoter 5mC **(A)** or 5hmC **(B)** patterns stratified into one of four groups: i = constitutively depleted in 5mC, ii = constitutively enriched in 5mC, iii = Sprague–Dawleyenriched 5mC, iv = Wistar-enriched 5mC. Heatmaps represent DNA modification levels over regions spanning the TSS ± 1 kb. Average patterns for each group are plotted on the right. **(C & D)** Visual examples of promoters belonging to groups ii, iii and iv for 5mC and 5hmC. 5mC: Methylated cytosine base; 5hmC: Hydroxymethylated cytosine base; TSS: Transcription start site.

**Figure 3:**
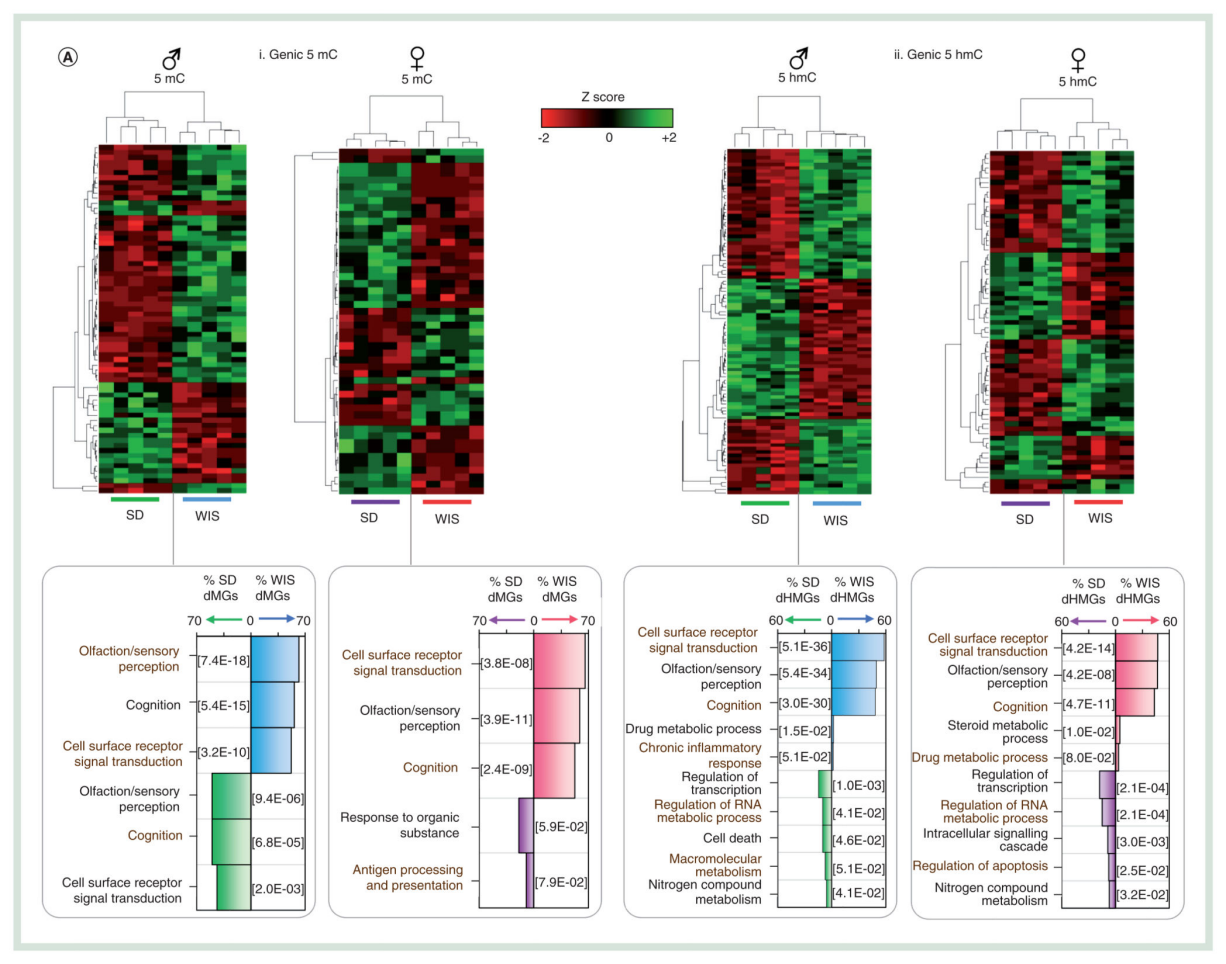

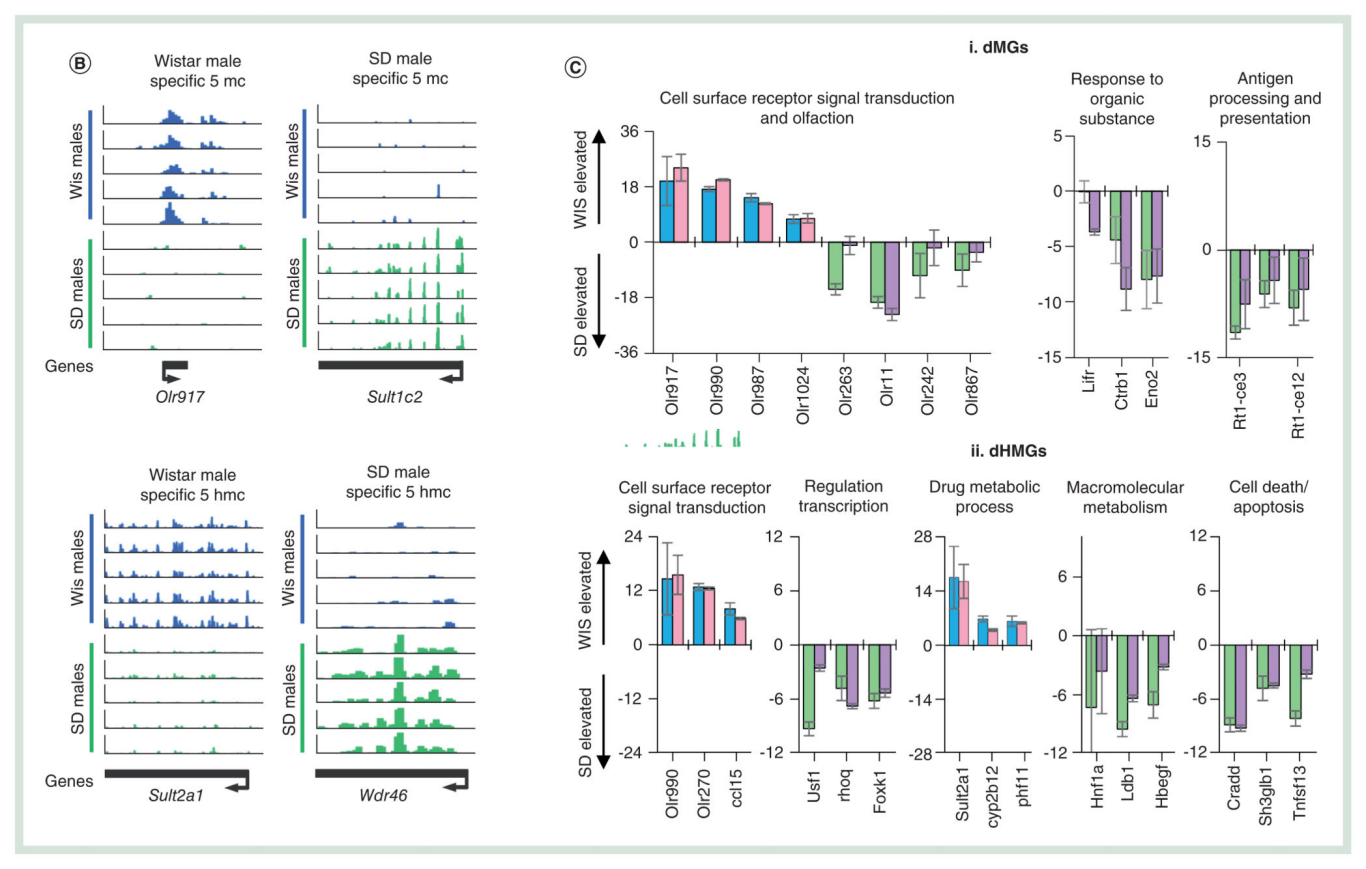
Chromatin modifications at enhancers and promoters. **(A)** Pearson correlation heatmaps with hierarchical clustering for H3K4me1 **(i)** and H3K27ac **(ii)** datasets. (**B)** Visual representation of genomic regions with either constitutive **(i)** or gender specific **(ii)** chromatin landscapes. **(C)** Box plot for H3K4me1 **(i)** and H3K27ac **(ii)** signals across one of six genomic compartments: promoter core: TSS ± 250 bp, promoter proximal: TSS +1 kb to +250 bp, Promoter distal: TSS +2 to +1 kb, exonic, intronic or intergenic. **(D)** Average patterns of H3K4me1 **(i)** and H3K27ac **(ii)** across annotated genes ±100% gene length. **(E)** Venn diagram of **(i)** H3K4me1 peak or H3K27ac **(ii)** overlaps between average male and female datasets. Square brackets denote total peak datasets. **(F)** Venn diagram of H3K4me1/H3K27ac peak overlaps for average male **(i)** and female **(ii)** datasets. Square brackets denote total peak datasets. **(G)** Average patterns of DNA modifications across either ‘poised’ **(i)** or active **(ii)** enhancer elements as well as at transcriptional start sites marked by similar chromatin marks **(iii & iv)**. H3K4me1: Histone H3 tails marked by lysine 4 monomethylation; H3K27ac: Histone H3 tails pan-acetylated at lysine 27; TSS: Transcription Start site.

**Figure 4:**
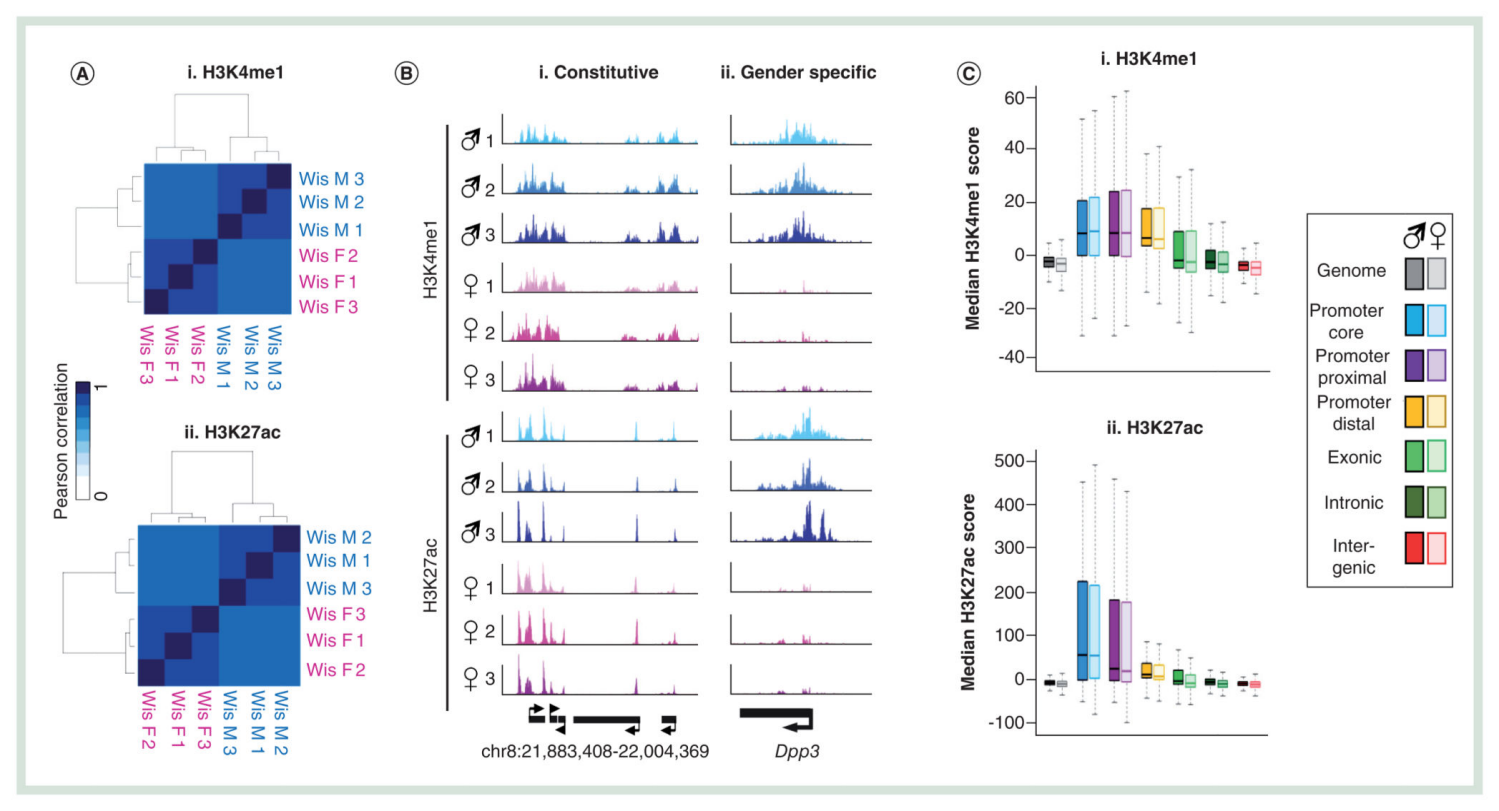

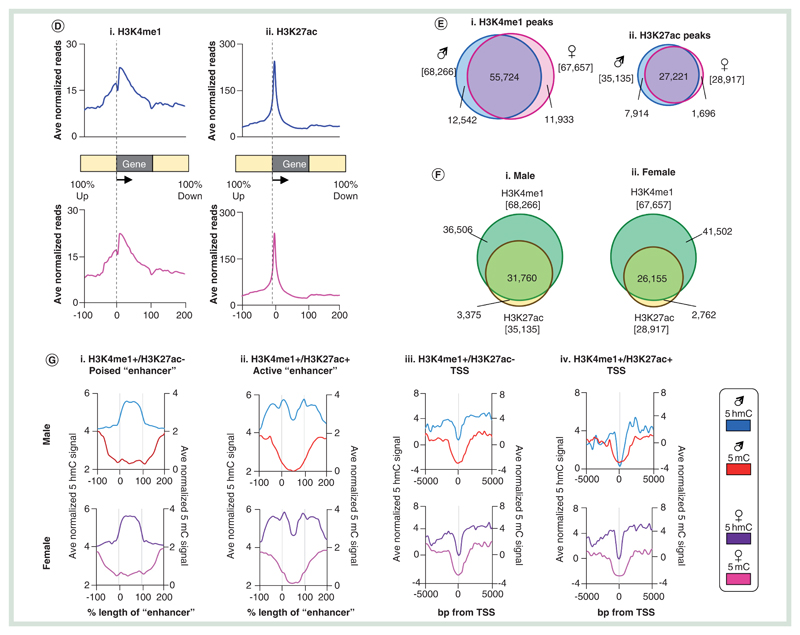
DNA modification patterns reflect the transcriptional state of the liver. **(A)** Average patterns of either 5mC **(i)**, 5hmC **(ii)**, H3K4me1 **(iii)** or H3K27ac **(iv)** over gene sets ±25% gene length, separated into quintiles based on published whole liver expression levels. All plots are produced from an average of male and female datasets. **(B)** Average male and female patterns of 5mC (top row), 5hmC (second row), H3K4me1 (third row) and H3K27ac (bottom row) across top 250 expressed housekeeping genes **(i)** or tissue-specific genes for liver **(ii)**, lung **(ii)**, kidney **(iii)** or heart **(iii)** using published datasets. Genic regions are highlighted in blue, promoters in yellow and upstream regulatory regions in green. Plots represent normalized gene lengths ±25%.

**Figure 5:**
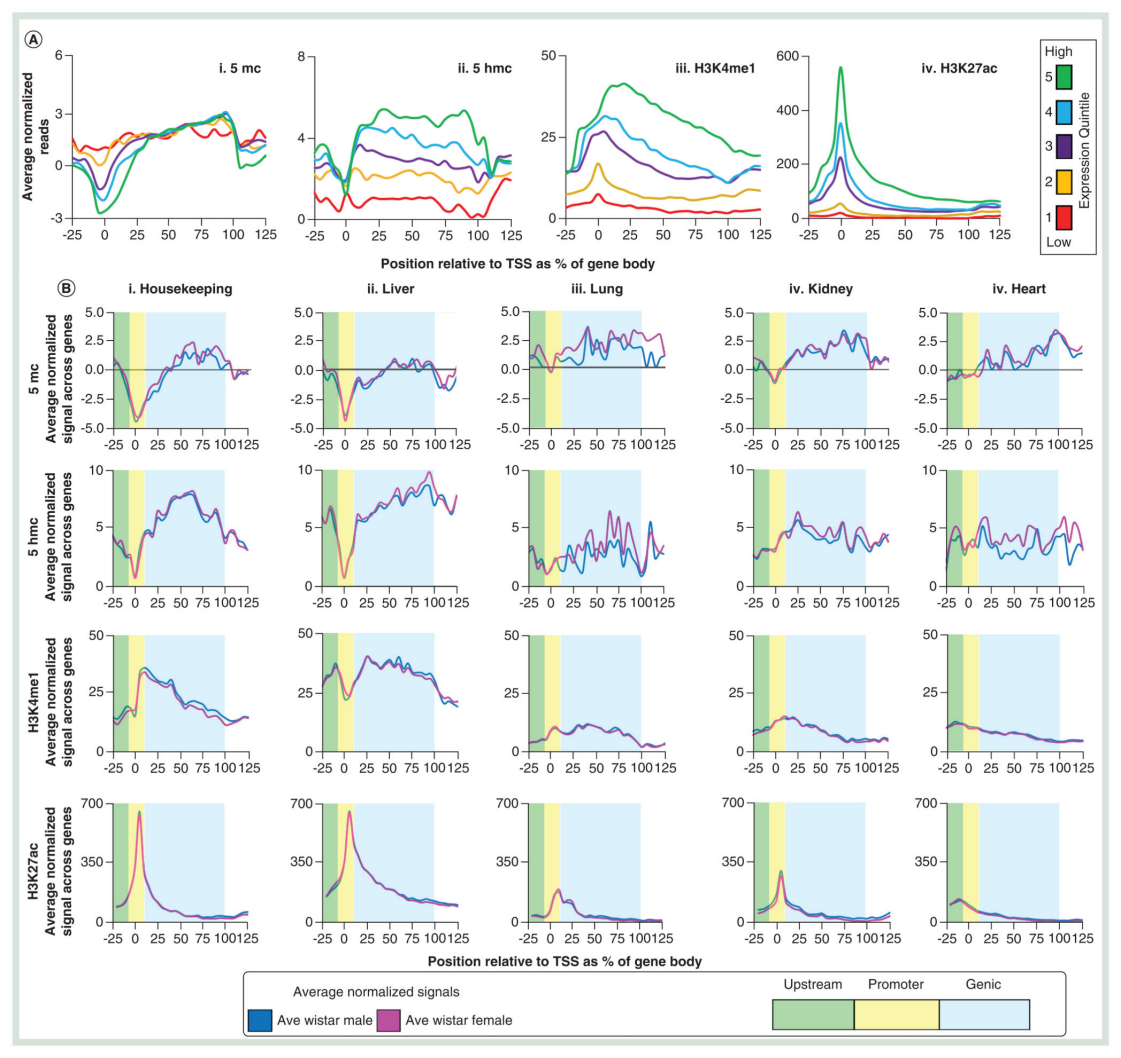
DNA modification and enhancer chromatin marks reflect transcriptional events. **(A)** Z-score heatmap for genes displaying strong gender-specific transcriptional bias. **(B)** GO term analysis of genes with gender-specific transcriptional bias. **(C)** Heatmap and average plots of differential gender epigenetic patterns over male-specific and female-specific genes. Blue bars/plots denote difference in signal male minus female signal while red denotes female-specific increase in signal. Average patterns highlight changes over total gene sets. Asterisk denotes TSS-specific changes in 5hmC signals at gender-specific genes. **(D)** Examples of epigenetic landscapes over male expressed **(i)**, female expressed **(ii)** or ubiquitously expressed **(iii)** genes. Gray box: upstream enhancer, yellow box: genic. Location of primers used for subsequent quantitative validation assays are represented below by blue arrows. **(E)** Bar chart of DNA modification levels at a given CpG within each of the regions highlighted by arrows in figure **(D)**. Plots show levels for three male and three female Wistar rat livers. Purple: % 5hmC, red: % 5mC, gray: % unmodified. All data representative of a single CpG dinucleotide within the sequence CCGG at each of the three loci. 5hmC: Hydroxymethylated cytosine base; TSS: Transcription start site.

Average patterns of each epigenetic mark were calculated across a series of genomic features, which were carried out using the Wellcome Trust Centre for Cell Biology Galaxy server tool ‘sliding window over length-normalized regions of interest’ [22]. In short, this function takes a set of genomic coordinates and calculates the patterns of DNA or histone modification from the supplied genome-wide data file in a series of windows. Regions: Figure 1F = Gene body ± 25% gene length, Figure 2B and Supplementary Figure 6 = TSS ± 1 kb, Figure 3D = Gene body ± 100% gene length, Figure 3G-i and -ii = Enhancer loci ± 100% enhancer length, Figure 3G-iii and -iv = TSS ± 5 kb, Figure 4A & B = Gene body ± 25% gene length, Figure 5C = Differential plot (difference between average male and female signals) across gene body ± 25% gene length.

#### Peak-finding & definition of differentially modified promoters & genes

**Figure 6:**
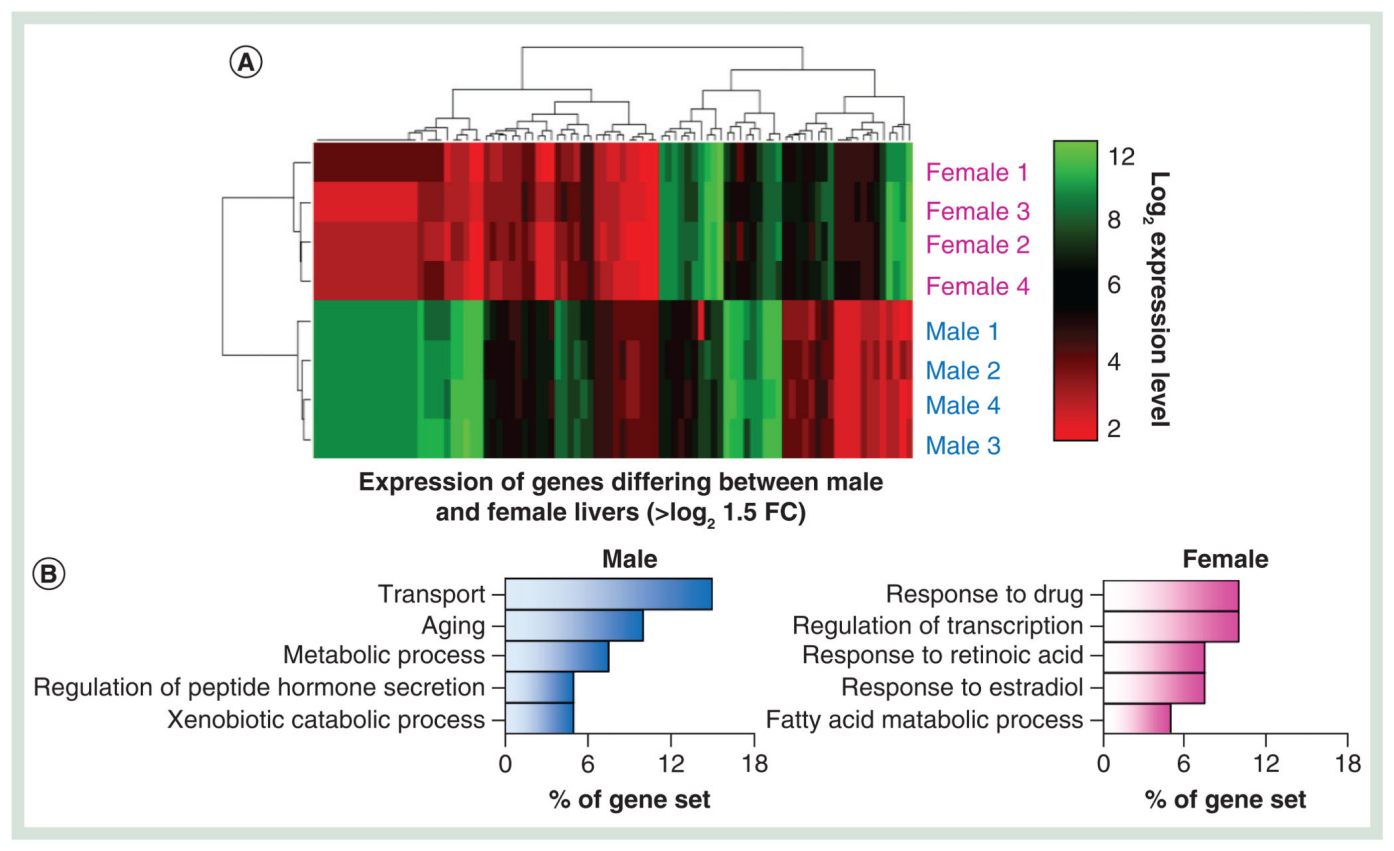

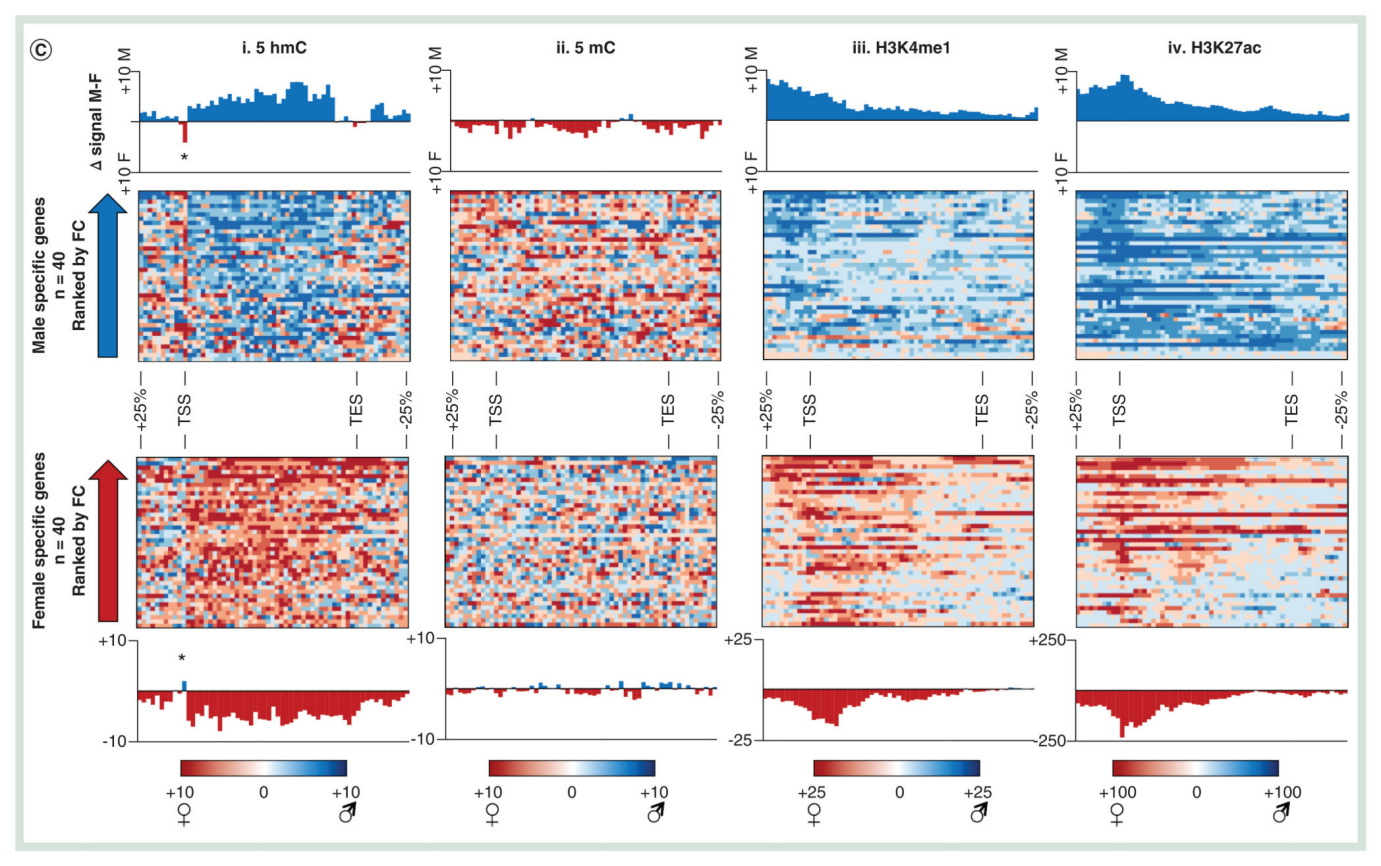

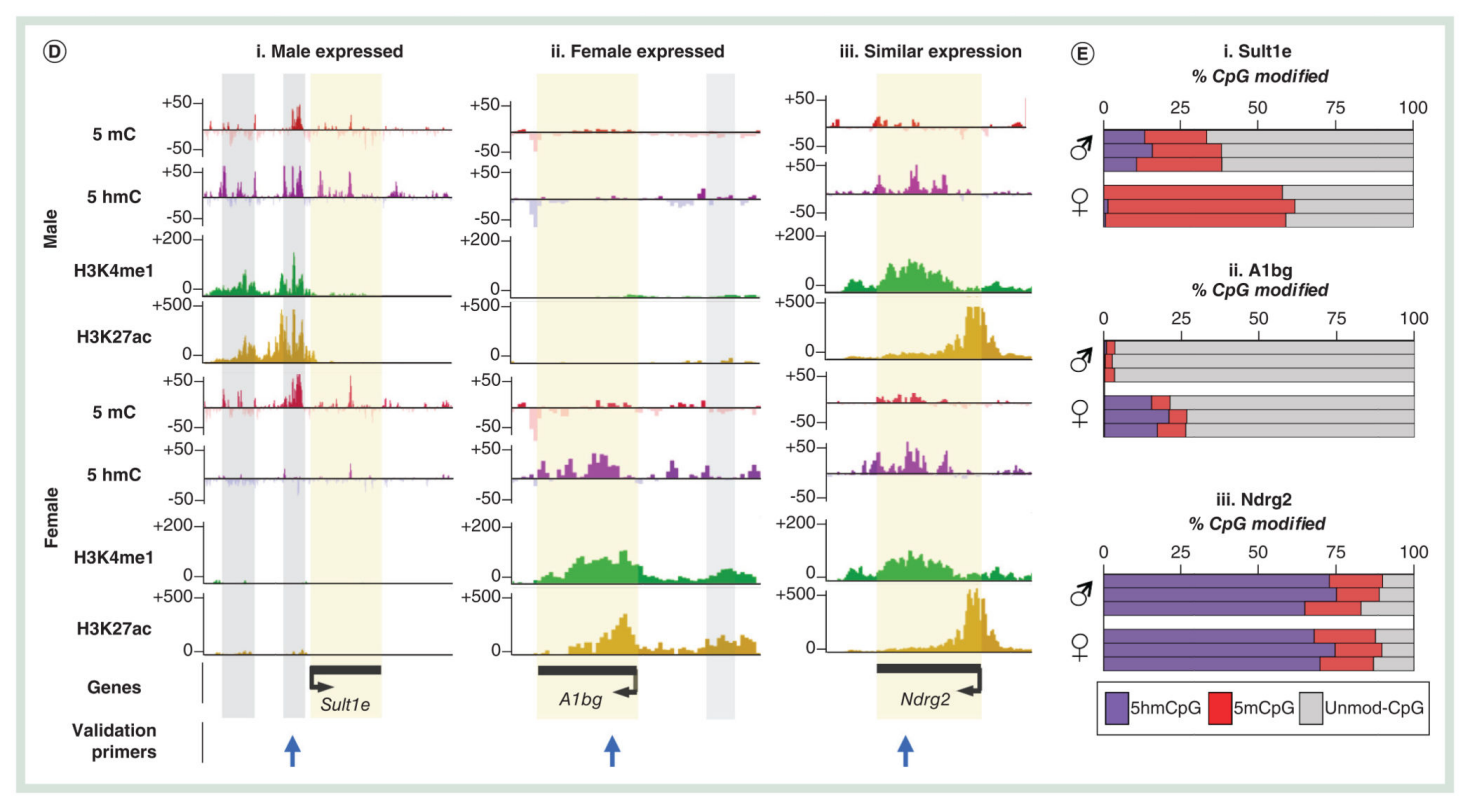
A number of gene bodies contain strain-dependent differences in their DNA modification levels. **(A)** Z-score heatmaps for average genic 5mC **(i)** and 5hmC **(ii)** levels over genes displaying strong reproducible strain-specific differences. dMG: strain differential methylated gene, dHMG: strain differential hydroxymethylated gene. Plots of GO terms are shown below. Plots represent percentage of dMGs or dHMGs in a given functional term (Wistar male: blue, Sprague–Dawley male green, Wistar female: pink, Sprague–Dawley female: purple). P-values associated with each functional term are plotted in square brackets. **(B & C)** Examples of strain-specific dMGs and dHMGs through visualization of patterns **(B)** or by stratification by functional gene class **(C)** extracted from the data produced in figure **(A)**. The differences in 5mC **(i)** or 5hmC **(ii)** between the averages of the two strains are plotted for each gene in figure **(C)**. 5mC: Methylated cytosine base; 5hmC: Hydroxymethylated cytosine base; dHMG: Differentially hydroxymethylated gene; dMG: Differentially methylated gene.

In short, peaks for DNA modifications were identified as described previously [10]. Peaks of chromatin marks were defined using the Wellcome Trust Centre for Cell Biology Galaxy server ‘peak finder’ tool with the following parameters: above 98th percentile of sequence read score and more than 300 bp long (= two binned windows of 150 bp). Poised enhancer elements were defined as genomic regions containing H3K4me1-enriched peaks within 5–1 kb of an annotated TSS. Similarly, active enhancers contain both H3K4me1 and H3K27ac peaks in these same regions. Promoters were defined as belonging to one of four groups (Figure 2) as follows: group i: average normalized and binned DNA modification score of less than 5 across TSS ± 250 bp in all samples, group ii: average normalized and binned DNA modification score of more than 5 across TSS ± 250 bp in all samples, group iii: average normalized and binned DNA modification score of more than 5 across TSS ± 250 bp in all Sprague–Dawley samples, group iv: average normalized and binned DNA modification score of more than 5 across TSS ± 250 bp in all Wistar samples. Genes changing in DNA modification levels between strains (Figure 6) were defined by calculating the average 5mC or 5hmC scores across gene body regions, which were enriched in all individuals of a given strain over the other by fivefold. Resulting differentially modified genes were then plotted by Z-score heatmap plots.

#### GO term functional analysis

Functional analysis of gene sets associated with differentially modified promoter or gene bodies was carried out using the GO TERM BP FAT feature on the DAVID functional annotation tool database [23].

Accession codes

We are currently in the process of uploading all raw and processed sequencing datasets at the Gene accession omnibus (GEO). Accession pending. Wistar male and female gene expression datasets can be found at GEO under the accession number GSE68128 and include datasets GSM1664281, GSM1664282, GSM1664283, GSM1664284, GSM1664289, GSM1664290, GSM1664291 and GSM1664292. Fischer 344 Rat RNAseq datasets are found at GEO under accession numbers GSM1328657, GSM1328641, GSM1328613, GSM1328598, GSM1328581, GSM1328565, GSM1328549 and GSM1328533.

## Results

### Global rat liver DNA modification patterns cluster by strain before gender

In order to generate ‘baseline’ epigenetic landscapes for liver of male and female Wistar and Sprague–Dawley rats, we employed a recently published method of next-generational semiconductor sequencing following antibody- based enrichment [24]. In short, we carried out DNA immunoprecipitation using specific antibodies to either 5mC or 5hmC on fragmented genomic liver DNA. These enriched libraries were then sequenced to approximately 35–40 million read depth on an Ion Proton semiconductor sequencer prior to bioinformatic processing including the binning of data into 150 bp windows and normalization to matched input samples (see Materials & methods section). Data were then plotted against the rat RN6 build and analyzed. In total, we generated genome-wide 5mC and 5hmC patterns for five male and five female rats, for Sprague–Dawley and Wistar strains each resulting in approximately 2.1 billion reads across 48 datasets (see Supplementary Table 1). Average 5mC and 5hmC datasets were also generated across the five replicates (Figure 1A). Datasets were initially validated by testing for reproducible enrichment or absence of modifications at a number of loci previously identified in the lab as acting as positive or negative control regions (Supplementary Figure 1). Subsequent generation of DNA modification peaks (sites of significant enrichment) reveals near equal 5hmC and 5mC enrichment across all chromosomes following length normalization (peaks/base pairs), with the exception of the sex chromosomes, which show strong gender-specific patterns of both 5mC and 5hmC (Supplementary Figure 2).

The relative enrichment levels for both 5mC and 5hmC were carried out across the genome and assigned to one of six compartments (promoter core: TSS ± 250 bp, promoter proximal: TSS +1 kb to +250 bp, promoter distal: TSS +2 to +1 kb, exonic, intronic or intragenic; Figure 1B). As had been previously reported for both mouse and human tissues, 5mC was found to be depleted over promoter elements and instead enriched in exonic and intragenic compartments, while 5hmC was enriched over promoter distal, exonic and intronic loci (Figure 1B) [25]. In both cases, the distributions of 5mC and 5hmC are similar between the two rat strains. Clustering of Pearson’ correlation values for each DNA modification dataset highlights the overall reproducibly of both 5mC and 5hmC patterns between all of the rats (cor range = 0.741–0.907 for 5mC, cor range = 0.704–0.932 for 5hmC; Figure 1C & D). Despite this, there is a clear stratification of individuals first by strain and then by gender (Figure 1C & Supplementary Figure 3). Overall variation within cohorts was typically very low for both 5mC (ave Pearson cor male Sprague–Dawley = 0.914, female Sprague–Dawley = 0.912, male Wistar = 0.901, female Wistar = 0.896) and 5hmC (ave Pearson cor male Sprague–Dawley = 0.946, female Sprague–Dawley = 0.953, male Wistar = 0.911, female Wistar = 0.893) and where present tended to occur in coding regions where the majority of the modification was found to reside and did not represent strong losses or gains at a given locus (Figure 1B, E & F).

Scatter plot analysis of 5hmC and 5mC signals between average datasets reveals that although sex chromosomes account for a portion of the variation between datasets, this is less than that observed at throughout the rest of the genome between strains (Supplementary Figure 3). For example, the correlation coefficients for 5mC between males and females of a given strain range from 0.927 to 0.905 for Wistar and Sprague–Dawley rats, respectively, while intrastrain cor values for a given gender range from 0.862 to 0.888 for Wistar versus Sprague–Dawley males and Wistar versus Sprague–Dawley females, respectively (Supplementary Figure 3). However, unlike the minimal variation observed between replicates of a given group (i.e., Wistar males), the majority of variation was seen to come from noncoding portions of the genome (Supplementary Figure 4) indicating that epigenetic differences observed between strains are linked to genotypic differences, the majority of which reside in noncoding DNA.

### A small number of promoter & genic DNA modification patterns differ between rat strains

As epigenetic modification of promoter regions has been linked to transcriptional activity from associated genes, we first set out to investigate 5mC and 5hmC patterns across these regions (see Materials & methods section; Supplementary Figure 5). Analysis of the rat liver epigenomes results in the stratification of promoters (TSS ± 1 kb) into one of four groups per modification. Group i represents the vast majority of promoters; those which are constitutively unmodified in all individuals (n = 18,350 5mC, n = 17,969 5hmC; Figure 2A–C). Contrastingly, a number of promoters are marked by modified CpGs in all individuals across both rat strains tested (n = 369 5mC, n = 764 5hmC; Figure 2A–C). A small number of promoters exhibit strong strain-dependent differences in 5mC (Sprague–Dawley specific: n = 16, Wistar specific: n = 30) and for 5hmC (Sprague–Dawley specific: n = 17, Wistar specific: n = 16). Interestingly, while 5hmC levels are typically also elevated at the 5mC marked promoters and vice versa, there is no clear relationship between the two marks at strain specific differentially modified promoters (Supplementary Figure 6). GO term analysis reveals that constitutively methylated promoters (5mC group ii) tend to be associated with functions such as cognition and detection of chemical stimuli while constitutively 5hmC-marked promoters (5hmC group ii) are associated with roles such as regulation of apoptosis, proliferation and response to hormones (data not shown). In addition, these 5hmC-marked promoters are strongly enriched for pathways involved in cancer progression, similar to observations for 5hmC-marked promoters in the mouse liver [12]. We did not detect a significant enrichment in the function of genes with strain-dependent differentially modified promoters (groups iii & iv).

Following on from the study of promoter regulatory regions, we next turned our attention to the DNA modification landscapes in genic portions of the genome. As described above, these regions tend to be enriched for both 5hmC and to some degree 5mC (Figure 1B) and exhibit the regions of greatest variance within groups of rats in a given strain and gender (Figure 1E). By comparison, a greater number of gene bodies are reproducibly altered between the two strains of rat (Figure 2A & Supplementary Figure 5). 5hmC levels are found perturbed over more gene bodies than 5mC (defined as >fivefold change in average genic DNA modification level in all five rats in a given group). Specifically, genic 5mC levels are reproducibly altered over 70 male genes (46 Wistar, 24 Sprague–Dawley elevated) and 62 female genes (32 Wistar, 30 Sprague–Dawley elevated; Supplementary Figure 5). By comparison, 438 genes exhibit reproducible 5hmC levels in male rats (149 Wistar, 289 Sprague–Dawley elevated) and 292 in female (72 Wistar, 220 Sprague–Dawley elevated; Figure 6A & Supplementary Figure 5). Strain-dependent epigenetic patterns over these genes were strong enough to clearly stratify groups of Sprague–Dawley and Wistar rats (Figure 6A & Supplementary Figure 7). Interestingly, only a proportion of differentially methylated genes (dMGs) or differentially hydroxymethylated genes (dHMGs) were observed both in male and females of a given strain (26% male dMGs and 29% female dMGs overlap, 22% male dHMGs and 33% female dHMGs overlap; Supplementary Figure 8A), highlighting the fact that there is not a common set of differentially modified genes between strains *per se* but that gender influences such strain-dependent differences. Furthermore, strain-specific changes to either 5mC or 5hmC were not typically found at the same gene sets, with only a minority of genes (n = 27 male, n = 20 female) found to contain both a dHMG and dMG across both strains (Supplementary Figure 8B). In almost all cases, these were cooperative changes in which strain-dependent elevation for 5hmC was accompanied by 5mC and vice versa. Functional analysis of the dMG and dHMG sets identified a number of significantly enriched GO terms. Typically, dMGs were associated with changes in genes with roles in olfaction, sensory perception and cognition (Figure 6A–C). In addition, there were a smaller but significant number of genes with roles relating to responses to organic substances, cell surface signal transduction and immune response (antigen processing and presentation; Figure 6A–C). Functional terms associated with dHMGs were far more diverse, possibly in part due to the larger number of genes exhibiting differential 5hmC levels. Similarly to the dMGs, a large proportion of the dHMGs were associated with cell surface signal transduction events (58% male, 47% female), olfaction (49% male, 47% female) and/or cognition (48% male, 43% female). Interestingly, a small yet significant proportion of genes were associated with regulation of transcription (14.6% male, 17.7% female), cell death/apoptosis (10% male, 7.5% female), macromolecular metabolism (7.3% male only), steroid metabolic processes (5% female only) or drug metabolic processes (2.3% male, 3.3% female). The differential epigenetic modification of genes associated with xenobiotic metabolism pathways in the baseline state between the two rat strains as well as across genders highlights potentially important functional differences in xenobiotic metabolism that warrants further biochemical validation and consideration for the interpretation of pharmacokinetic assessments supporting rat toxicology studies.

### Enhancer chromatin modifications & transcriptional environments display unique affiliations with 5hmC & 5mC-enriched DNA

It has previously been shown that 5hmC is particularly enriched over enhancer elements in mouse and human cells and tissues [26–29]. As such loci represent regions of particular interest toward the establishment of transcriptional landscapes in the liver and may act as potential targets for xenobiotic compounds we set out to characterize these between genders in a number of Wistar rats. Genome-wide chromatin immunoprecipitation sequencing (ChIP-seq) was carried out for histone H3 tails marked by either lysine 4 monomethylation (H3K4me1) or pan-acetylated at lysine 27 (H3K27ac) to identify both ‘poised’ (H3K4me1+ve/H3K27ac-ve) and ‘active’ (H3K4me1+ve/H3K27ac+ve) enhancer elements [30]. Clustering of Pearson’ correlation values for either H3K4me1 or H3K27ac highlights both the overall high level of reproducibility across samples, particularly between individuals of a given gender (mean Pearson cor values: male H3K4me1 = 0.930, female H3K4me1 = 0.951, male H3K27ac = 0.949, female H3K27ac = 0.962; Figure 3A & B). Mapping the genomic distributions to one of the six compartments described above (Figure 1B) reveals relatively similar patterns for H3K4me1 and H3K27ac modifications with strong enrichments for both found in promoter core and proximal regions alongside moderate levels of enrichment over promoter distal sites (Figure 3C & D & Supplementary Figure 9). In addition, H3K4me1-modified histone tails are also found enriched somewhat in exonic regions.

Next, we carried out peak finding on the chromatin modification datasets in order to define regions reproducibly marked between males and females as well as those marked by H3K4me1 alone or by both marks (see Materials & methods section). 81.6% of male H3K4me1 peaks and 82.4% female peaks of H3K4me1 were found to directly overlap between the genders while 77.4% of male H3K27ac peaks and 94.1% of female H3K27ac peaks overlapped between the two genders (Figure 3E). We find that although the majority H3K27ac peaks overlap with a peak of H3K4me1 (90.4% for both males and females, respectively), a large number of H3K4me1 only peaks were also present (53.5% of male and 61.3% of female H3K4me1 peaks; Figure 3F). Due to the relative enrichment across promoter proximal and distal loci, these two populations will in part represent both ‘poised’ (H3K4me1+ve/H3K27ac-ve) and ‘active’ (H3K4me1+ve/H3K27ac+ve) enhancer elements. Previous studies in mouse and human tissues have identified a unique DNA modification landscape across such enhancer elements [28,31–33]. As such we set out to define the 5mC and 5hmC landscapes across poised and active enhancers in the rat over sets of peaks, which are either associated with a TSS or regions defined as ‘enhancers’ due to their proximity to nearby genes (within a 5-kb window upstream of an annotated gene). There is a strong depletion of 5mC over both enhancer elements (both at ‘poised’ and ‘active enhancers’) and transcriptional start sites in the rat liver (Figure 3G). Contrastingly, in the case of 5hmC, genomic context appears to play a fundamental role, with 5hmC strongly enriched over the core of ‘poised’ enhancers and in the flanks of ‘active’ enhancers but instead depleted over transcriptional start sites containing the same combinations of histone tail modifications (Figure 3G).

Next, we looked to test for relationships between DNA modification and chromatin modification states to the transcriptional activities of annotated rat genes. Comparisons of our Wistar rat 5mC, 5hmC, H3K4me1 and H3K27ac patterns to published Wistar rat gene expression datasets reveal clear relationships between promoter and genic epigenetic landscapes and transcriptional output following stratification into expression quintile groups (Figure 4A) [20]. In a similar manner to mouse and human studies, highly expressed genes tend to contain low levels of promoter 5mC and 5hmC and high levels of both H3K4me1 and H3K27ac (Figure 4A). Regions upstream of highly transcribed genes are typically devoid of 5mC-marked DNA and instead enriched for 5hmC and both H3K4me1 and H3K27ac-marked chromatin (possibly relating to enhancer elements). Gene bodies of highly transcribed genes were strongly enriched for 5hmC and 5mC as well as for the H3K4me1 modification. Contrastingly genes with reduced transcriptional output exhibited progressively elevated levels of promoter 5mC and 5hmC modification alongside a reduced level of H3K4me1 and H3K27ac histone tail modification. Upstream elements associated with lowly transcribed genes tended to contain higher levels of 5mC and lower levels of 5hmC, H3K4me1 and H3K27ac. Stratification of gene sets based on tissue-specific expression patterns further reveals unique patterns of both DNA and histone tail modifications across either housekeeping genes, liver, lung, kidney or heart-specific genes [21]. Using our liver epigenetic datasets, we find that at both housekeeping and liver-specific genes, 5mC and 5hmC are typically depleted from promoter sites while H3K4me1 and H3K27ac are enriched. 5hmC patterns and H3K4me1 patterns are also strongly enriched in the gene bodies of these same gene sets. Contrastingly in the liver, both 5mC and 5hmC levels are higher and H3K4me1 and H3K27ac levels are lower over the promoters of lung, kidney and heart-specific genes; resulting in defined modification landscapes for housekeeping and liver-specific genes compared with silent genes.

Together these results indicate that the rat liver transcriptome is strongly related to the epigenetic landscapes across both the coding and noncoding portions of genes and can potentially highlight the sensitivity of epigenetic profiling where transcription is altered (i.e., following exposure to chemical agents) [10].

### A series of combined chromatin changes reflect gender-specific transcriptional changes

Xenobiotic exposure frequently results in a number of strong transcriptional changes such as rapid changes in the levels of CYP450 and general detoxification genes. Xenobiotic-induced changes to the epigenetic landscapes may in part regulate changes in transcriptional activities at a number of genes, particularly through changes in the DNA modification and histone tail modification patterns at promoter and enhancer loci [10,15]. To investigate the robustness of these epigenetic-based assays in reporting on differentially expressed genes, we compared the epigenetic landscapes between male and female Wistar rats – focusing on genes exhibiting strong gender transcriptional bias (>log2 1.5-fold change in gene expression across all individuals of a given gender; n = 4, Figure 5A & B & Supplementary Figure 10). Closer inspection of these genes reveals a unique chromatin landscape at these loci both at upstream, promoter core and genic regions (Figure 5C & D). At gender-specific genes, there is typically a lower amount of 5hmC over the promoter core region and greater levels of upstream and genic 5hmC levels where the gene is active (asterisk Figure 5C) – again in agreement with the relationships with transcriptional activity described earlier (Figure 4A & B). In contrast, 5mC changes are less obvious across active and inactive gender-specific genes (Figure 5C & D). Finally, both H3K4me1 and H3k27ac modifications are clearly enriched over promoter core, genic and upstream loci across actively transcribing gender-specific genes (Figure 5C & D). Independent validation of loci displaying gender-specific epigenetic patterns using a quantitative enzymaticbased approach (see Materials & methods section) both confirm the results of the sequencing analysis as well as highlight the low intraindividual variance within individuals of a given strain (Figure 5E). Together, these results highlight the utility of combined 5hmC/’enhancer chromatin mark’-based analysis over the analysis of the more traditionally studied 5mC modification for inclusion in future hepatotoxicity mechanistic studies.

## Discussion

One significant challenge associated with interpreting xenobiotic interactions with the epigenome is the need to characterize the normal interindividual tissue- and cell-type-specific dynamics of epigenetic modifications within healthy, injured and diseased tissues. These baseline data are critically important for interpreting adverse versus adaptive epigenetic changes. Furthermore, there is also a need to assess the translatability of xenobiotic-induced epigenetic perturbations across strains and species in order to assess human relevance. Here, we describe a liver tissue-specific epigenome resource for two laboratory rat strains that are commonly used for safety assessment of chemical and therapeutic drugs. Epigenetic modifications are thought to both reflect and in part regulate the transcriptional output of a given cell and may act as a novel method to test for the early events associated with hepatotoxicity and hepatocarcinogenesis [6]. Previously, we have reported by analysis of promoter-specific microarrays that the study of the DNA modifications 5mC and 5hmC reflects toxicological insult in mouse livers following exposure to the rodent nongenotoxic carcinogen, PB [10,14–15]. Such studies have provided a number of early-stage biomarkers for PB-related carcinogenesis as well as providing a novel insight into the molecular mechanism associated with nongenotoxic carcinogenesis [11,16,34–35]. However, in order to fully exploit the potential application of such epigenetic-based research in these sectors, it is vital that we employ new genome-wide approaches to fully characterize the epigenomes in a number of toxicologically relevant animals. In this study, we have generated the first baseline genome-wide DNA modification landscape maps for 5mC and 5hmC across the liver of both male and female Sprague–Dawley and Wistar rat strains. These two strains of rats are routinely used for in vivo toxicity testing of xenobiotics. We find that genome-wide patterns of both 5mC and 5hmC are reproducible between individuals of a given strain and gender and cluster first by strain and then gender (i.e., Wistar males and females are more similar than Wistar males and Sprague–Dawley males). Where present intragroup variation (i.e., between sets of Wistar females) occurred within coding portions of the genome while intergroup variation (i.e., between Wistar males and Sprague–Dawley males) were found in noncoding portions of the genome – possibly due to differences in genotype. Although variation was minor, we identified a number of promoter elements and gene bodies displaying strong strain-dependent DNA modification differences (Figures 2 & Figure 6). Interestingly, a number of these loci were associated with genes with roles such as drug metabolism, steroid metabolism and cell surface receptor signaling (Figure 6A). Expansion of the analysis to investigate chromatin marks linked with functionally important enhancer elements also reveals low levels of intrastrain variation (Figure 3). Finally, by focusing on a number of genes displaying gender-dependent expression patterns, we highlight the utility of combined 5hmC/H3K4me1/H3K27ac profiling to reflect transcriptional state. The interrogation and integration of genome-wide liver epigenetic states alongside expression profiling studies has the potential to identify unique epigenetic signatures for diverse drug modes of action and toxicity pathways. These studies and methodologies can enhance the mechanistic understanding of xenobiotic exposure events and can also enable the identification of novel safety biomarkers. It is noteworthy that fibrotic pathology in nonalcoholic fatty liver disease has recently been shown to be associated with gene-specific cell-free plasma DNA methylation biomarkers [36], thus raising the possibility of bridging xenobiotic-induced perturbations of the rat liver epigenome to peripheral epigenetic biomarkers.

## Supplementary Material

To view the supplementary data that accompany this paper please visit the journal website at: www.futuremedicine.com/doi/full/10.2217/epi-2017-0029

Supplemental Materials

## Figures and Tables

**Table 1:** Datasets generated in this study.

ID	Strain	Gender	Animal #	Modification	# of reads
1	Sprague–Dawley	Male	1	5mC	38086812
2	Sprague–Dawley	Male	2	5mC	35841200
3	Sprague–Dawley	Male	3	5mC	35546933
4	Sprague–Dawley	Male	4	5mC	39122844
5	Sprague–Dawley	Male	5	5mC	40623332
6	Sprague–Dawley	Female	1	5mC	35790165
7	Sprague–Dawley	Female	2	5mC	42662832
8	Sprague–Dawley	Female	3	5mC	36547595
9	Sprague–Dawley	Female	4	5mC	34871466
10	Sprague–Dawley	Female	5	5mC	38834932
11	Wistar	Male	1	5mC	36081271
12	Wistar	Male	2	5mC	50813339
13	Wistar	Male	3	5mC	47275111
14	Wistar	Male	4	5mC	37969199
15	Wistar	Male	5	5mC	39355128
16	Wistar	Female	1	5mC	44566166
17	Wistar	Female	2	5mC	31658423
18	Wistar	Female	3	5mC	37892712
19	Wistar	Female	4	5mC	44208673
20	Wistar	Female	5	5mC	48270797
21	Sprague–Dawley	Male	1	5hmC	38876978
22	Sprague–Dawley	Male	2	5hmC	43082008
23	Sprague–Dawley	Male	3	5hmC	40164646
24	Sprague–Dawley	Male	4	5hmC	43628752
25	Sprague–Dawley	Male	5	5hmC	39309200
26	Sprague–Dawley	Female	1	5hmC	40450592
27	Sprague–Dawley	Female	2	5hmC	35422964
28	Sprague–Dawley	Female	3	5hmC	35823324
29	Sprague–Dawley	Female	4	5hmC	33966093
30	Sprague–Dawley	Female	5	5hmC	36771734
31	Wistar	Male	1	5hmC	42212574
32	Wistar	Male	2	5hmC	34357649
33	Wistar	Male	3	5hmC	44502144
34	Wistar	Male	4	5hmC	55553975
35	Wistar	Male	5	5hmC	34691960
36	Wistar	Female	1	5hmC	34712446
37	Wistar	Female	2	5hmC	43656067
38	Wistar	Female	3	5hmC	40578297
39	Wistar	Female	4	5hmC	29979987
40	Wistar	Female	5	5hmC	46246950
41	Sprague–Dawley	Male	1	INPUT	47726219
42	Sprague–Dawley	Male	2	INPUT	39445197
43	Sprague–Dawley	Female	1	INPUT	33736240
44	Sprague–Dawley	Female	2	INPUT	33260984
45	Wistar	Male	1	INPUT	55074883
46	Wistar	Male	2	INPUT	49077780
47	Wistar	Female	1	INPUT	32800318
48	Wistar	Female	2	INPUT	24895506
49	Wistar	Male	1	H3K4me1	29000000
50	Wistar	Male	2	H3K4me1	31000000
51	Wistar	Male	3	H3K4me1	32000000
52	Wistar	Female	1	H3K4me1	30000000
52	Wistar	Female	2	H3K4me1	30000000
54	Wistar	Female	3	H3K4me1	32000000
55	Wistar	Male	1	H3K27ac	45000000
56	Wistar	Male	2	H3K27ac	41000000
57	Wistar	Male	3	H3K27ac	42000000
58	Wistar	Female	1	H3K27ac	47000000
59	Wistar	Female	2	H3K27ac	51000000
60	Wistar	Female	3	H3K27ac	40000000

## Summary points

- In this study we generate the first genome-wide maps for DNAmodification states (5-methylcytosine & 5-hydroxymethylcytosine) and enhancer linked histone modification patterns (Histone H3K4 mono methylation & H3K27 pan-acetylation) in the livers of male and female Wistar and Sprague Dawley rats
- Analysis of the global DNA modification patterns reveal that these cluster by strain before gender.
- A small number of promoter & genic DNA modification patterns differ between rat strains. These typically relate to genes with roles in a range of pathways including those associated with drug metabolic processes and cell surface receptor signalling pathways and may be relevant in explaining difference in toxicological responses between rat strains
- Enhancer chromatin modifications & transcriptional environmentsdisplay unique affiliations with 5hmC & 5mC-enriched DNA.
- Together, a series of combined chromatin changes (5hmC, 5mC<H3K4me1, H3K27ac) reflect gender-specific transcriptional changes – highlighting utility towards future chemical safety assessment screens.
