# Supplementary material for: Defining baseline epigenetic landscapes in the rat liver
Source: Epigenomics. Author manuscript; Available in PMC 2018 May 17. (PMC5957268; doi:10.2217/epi-2017-0029)
Supplement: Supplemental Materials [file NIHMS77208-supplement-Supplemental_Materials.docx]

**Supplemental material: Defining the baseline DNA modification landscapes in the rat liver**

**John P Thomson, Raffaele Ottaviano, Roland Buesen , Jonathan G Moggs, Michael Schwarz and Richard R Meehan**

| **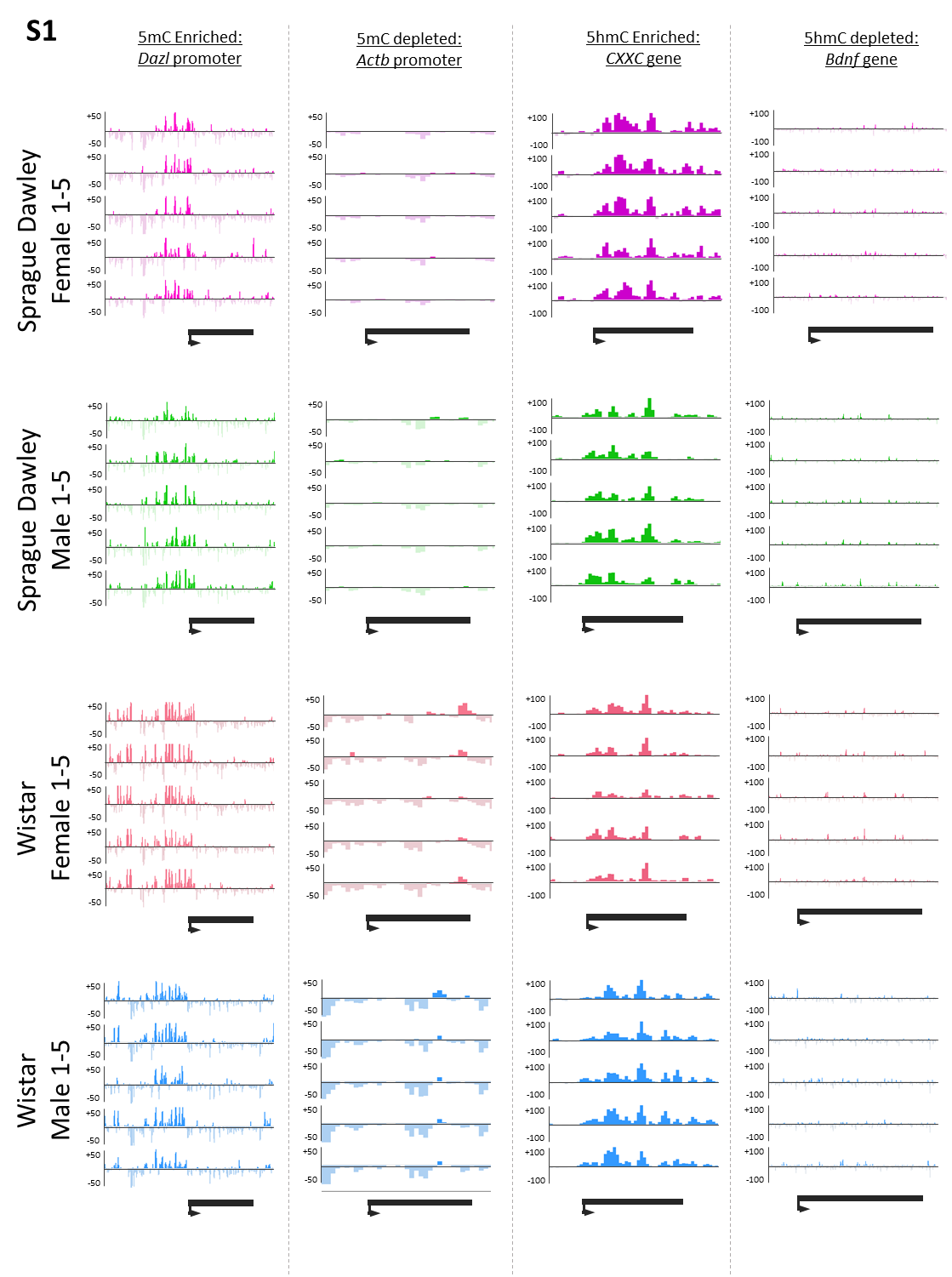** | **Figure S1.** Examples of sequencing data across predicted 5mC and 5hmC enriched and depleted loci. 5mC enriched locus over the promoter of the methylation regulated gene *Dazl*, 5mC depleted locus at the CpG island *Actb*, 5hmC enrichment over the gene body of the strongly expressed gene CXXC1 and 5hmC depletion over the brain specific gene *Bdnf* are all validated with all individuals of either genders of rat strains. |
| --- | --- |

| **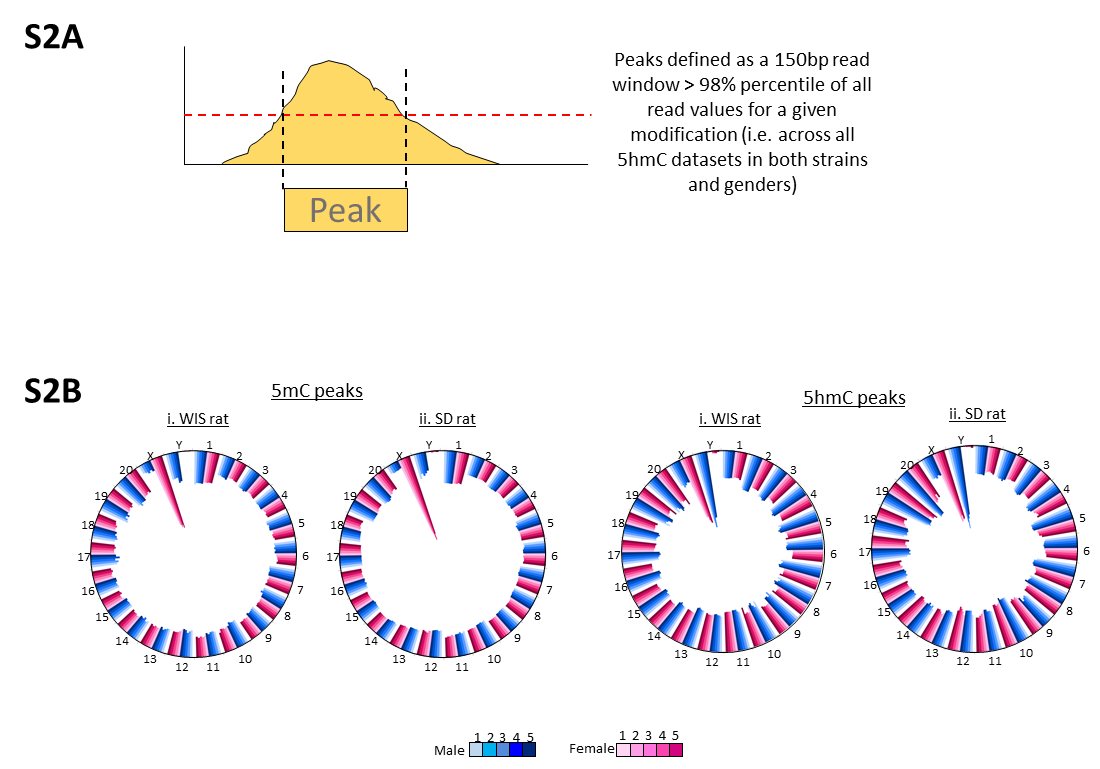** |
| --- |
| **Figure S2. A.** Schematic outlining how peaks are defined from the genome-wide datasets. See materials and methods for more information. **B**. Number of 5mC and 5hmC peaks per chromosome following length normalisation (peaks / bp). Males peaks: blues, female peaks: pinks. |

| **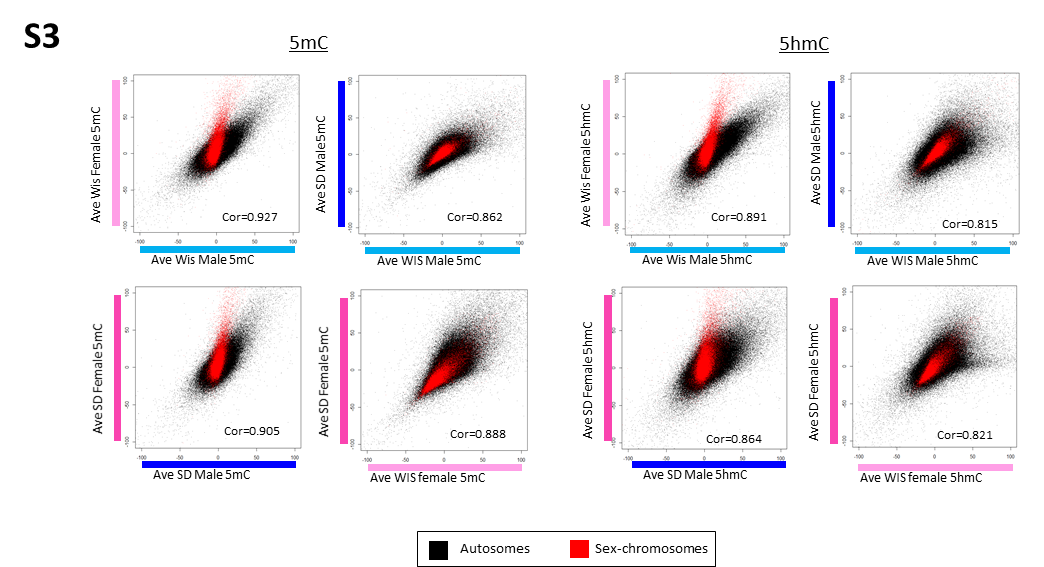** |
| --- |
| **Figure S3.** Scatter plots for average binned windows (average reads per 150bp) for genome wide 5mC and 5hmC datasets within and between strains and genders. Correlation values are shown within the plot. Black dots = autosomes, red dots = sex chromosomes. |

| **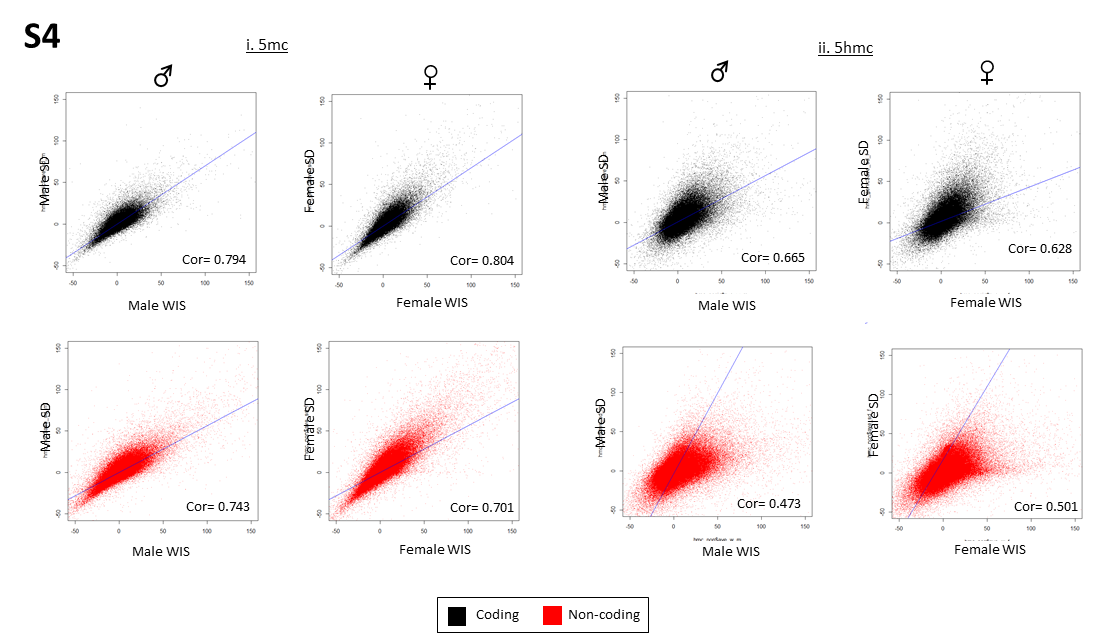** |
| --- |
| **Figure S4.** Scatter plots for average binned windows (average reads per 150bp) for genome wide 5mC and 5hmC datasets within and between strains and genders. Correlation values are shown within the plot. Black plots = coding portions of the genome (i.e. exonic and intronic) , red plots = non-coding loci. |

| **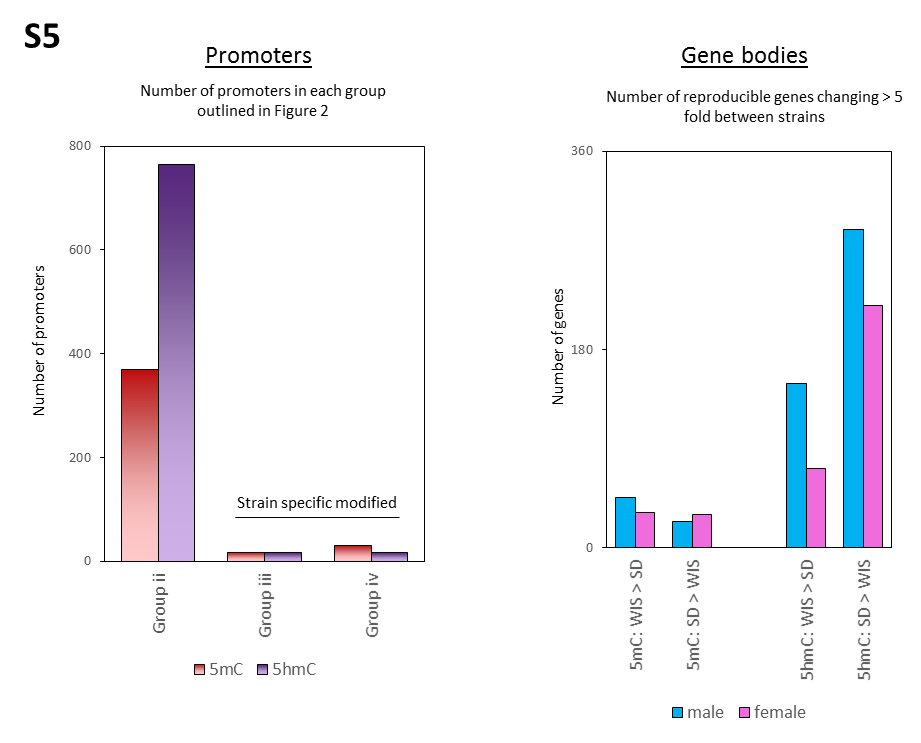** | **Figure S5.** Graphs relating to numbers of promoters in Figure 2 and gene bodies in Figure 3. Promoters are broken into 3 sets referring to groups ii (DNA mod enriched in all strains) , iii (Sprague Dawley enriched) and iv (Wistar enriched) from figures 2A & 2B. 5mC =red, 5hmC = purple. Number of gene bodies exhibiting strong strain specific epigenetic changes are shown on the right. Blue = male, pink = female. |
| --- | --- |

| **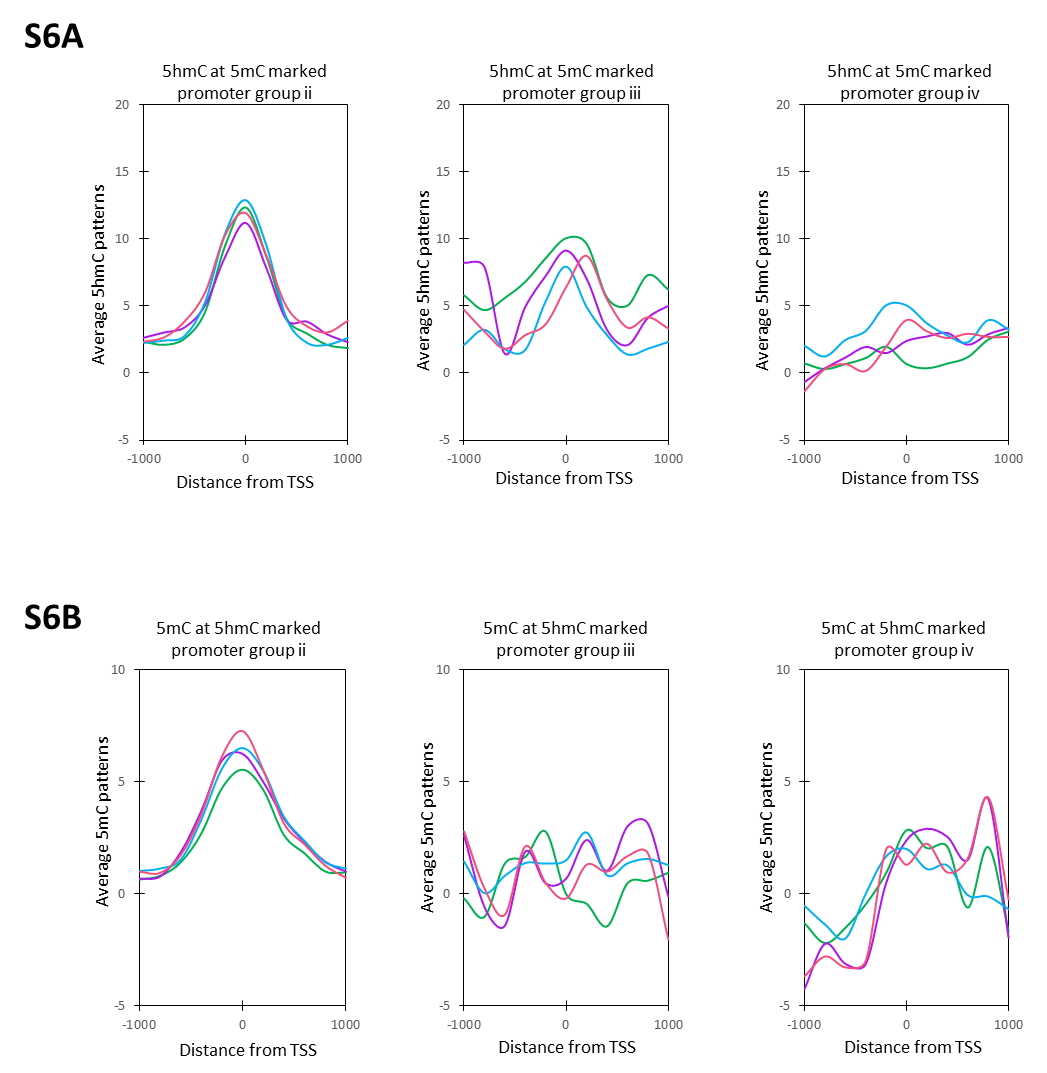** | **Figure S6.** Average 5hmC (**A**) and 5mC (**B**) levels across promoter groups from Figure 2A. Plots display average DNA modification patterns across loci identified in groups ii, iii and iv from figures 2A & 2B plotted through sliding window analysis (materials and methods). Blue: average Wistar male, pink: average Wistar female, green: average Sprague Dawley male, purple: average Sprague Dawley female. |
| --- | --- |

| **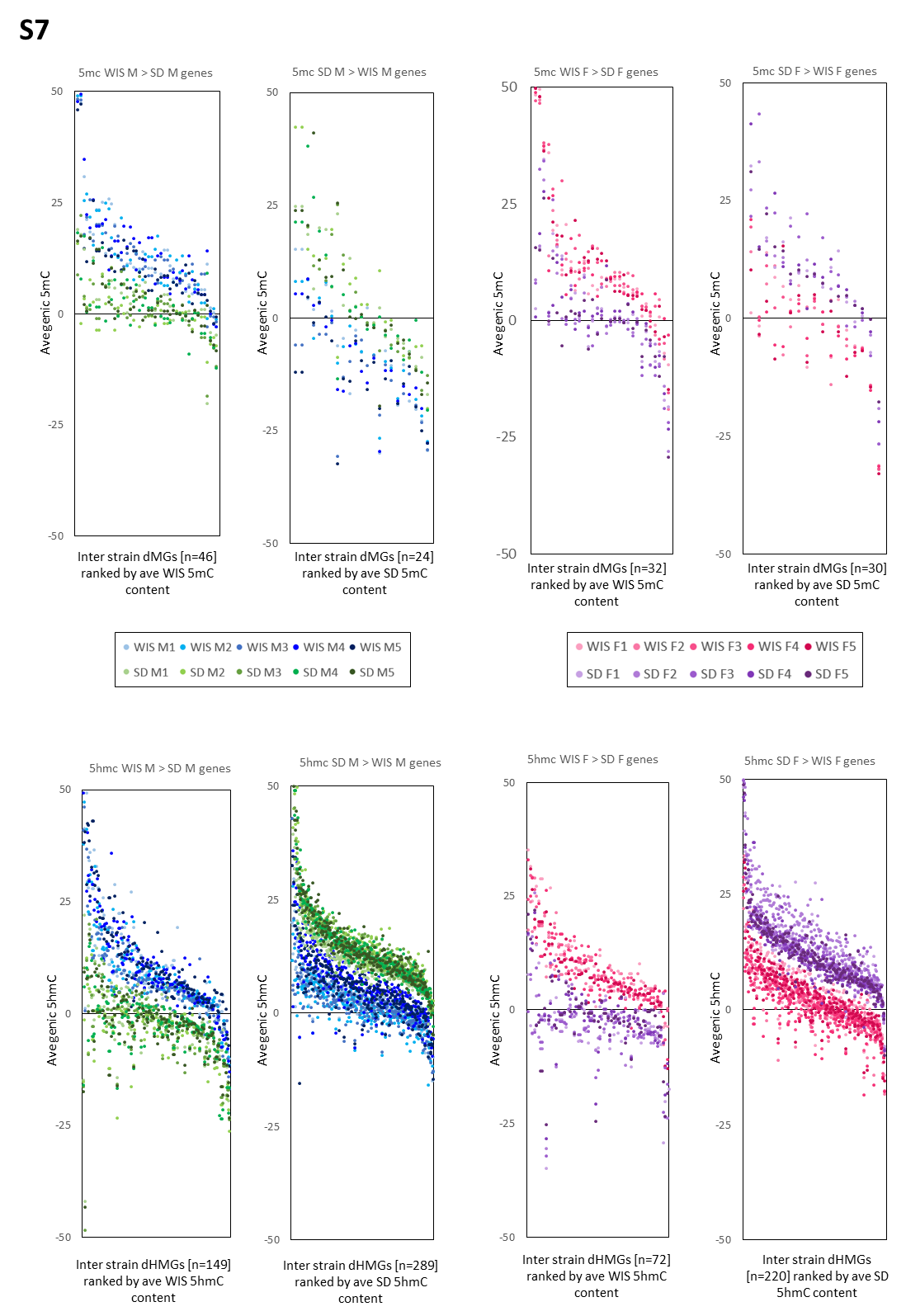** |
| --- |
| **Figure S7.** Levels of individual liver 5mC (Top rows) or 5hmC (bottom rows) across the dMGs and dHMGs identified in figure 3A. Loci are ranked by either average Wistar or Sprague Dawley levels. |

| **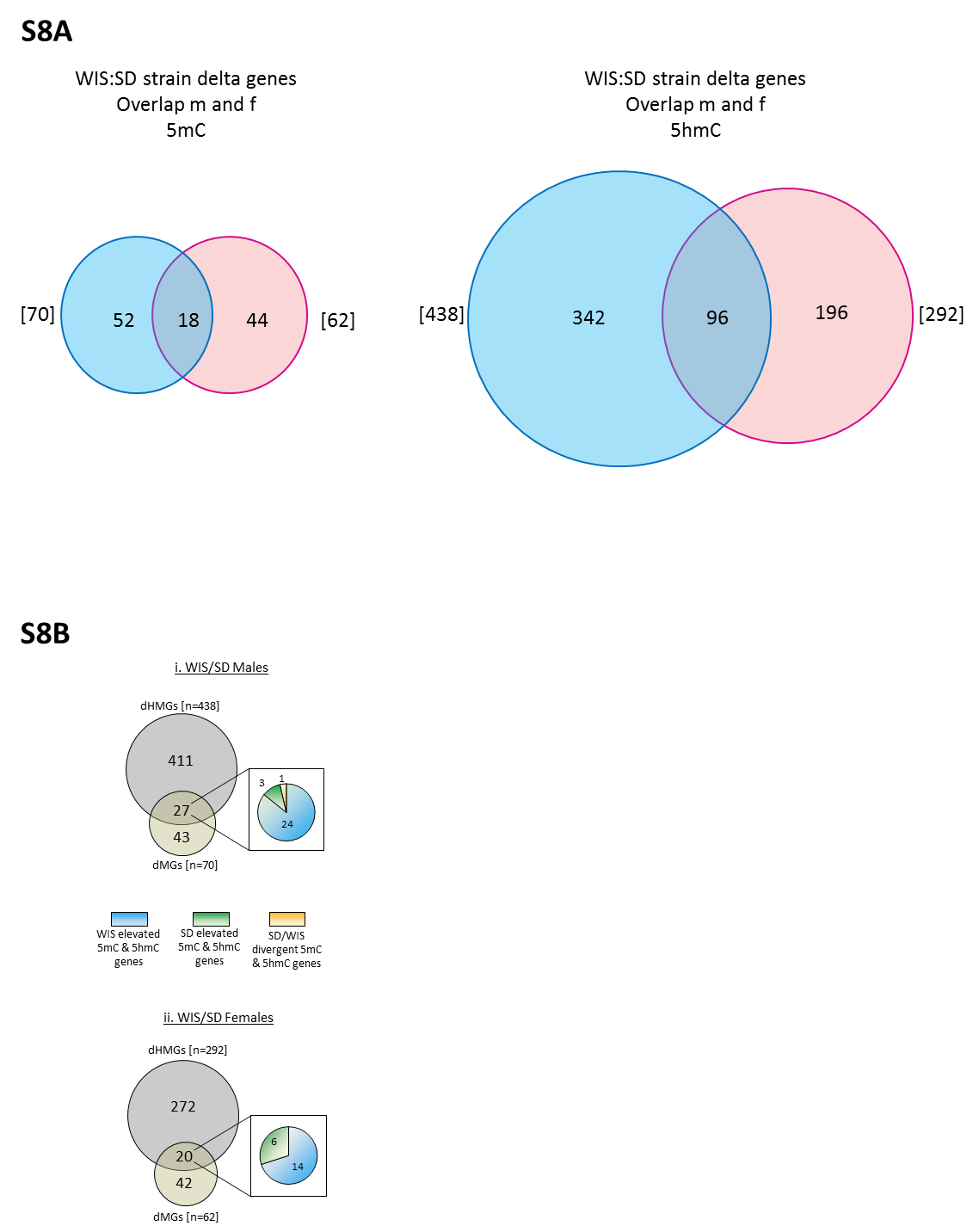** |
| --- |
| **Figure S8. A.** Venn diagram displaying the overlap of strain specific differentially methylated (left) or hydroxymethylated (right) genes between male and female livers. Male: blue, female: pink. Total number of differentially methylated or hydroxymethylated genes are represented by square brackets. **B.** Venn diagrams displaying the overlap of differentially methylated and hydroxymethylated genes by gender. Grey = dHMGs, gold= dMGs. Where overlap is observed (i.e. gene with both a change in 5mC and 5hmC) pie charts display further information (i.e. both 5hmC and 5mC elevated in a given strain or divergent within a strain). |
| **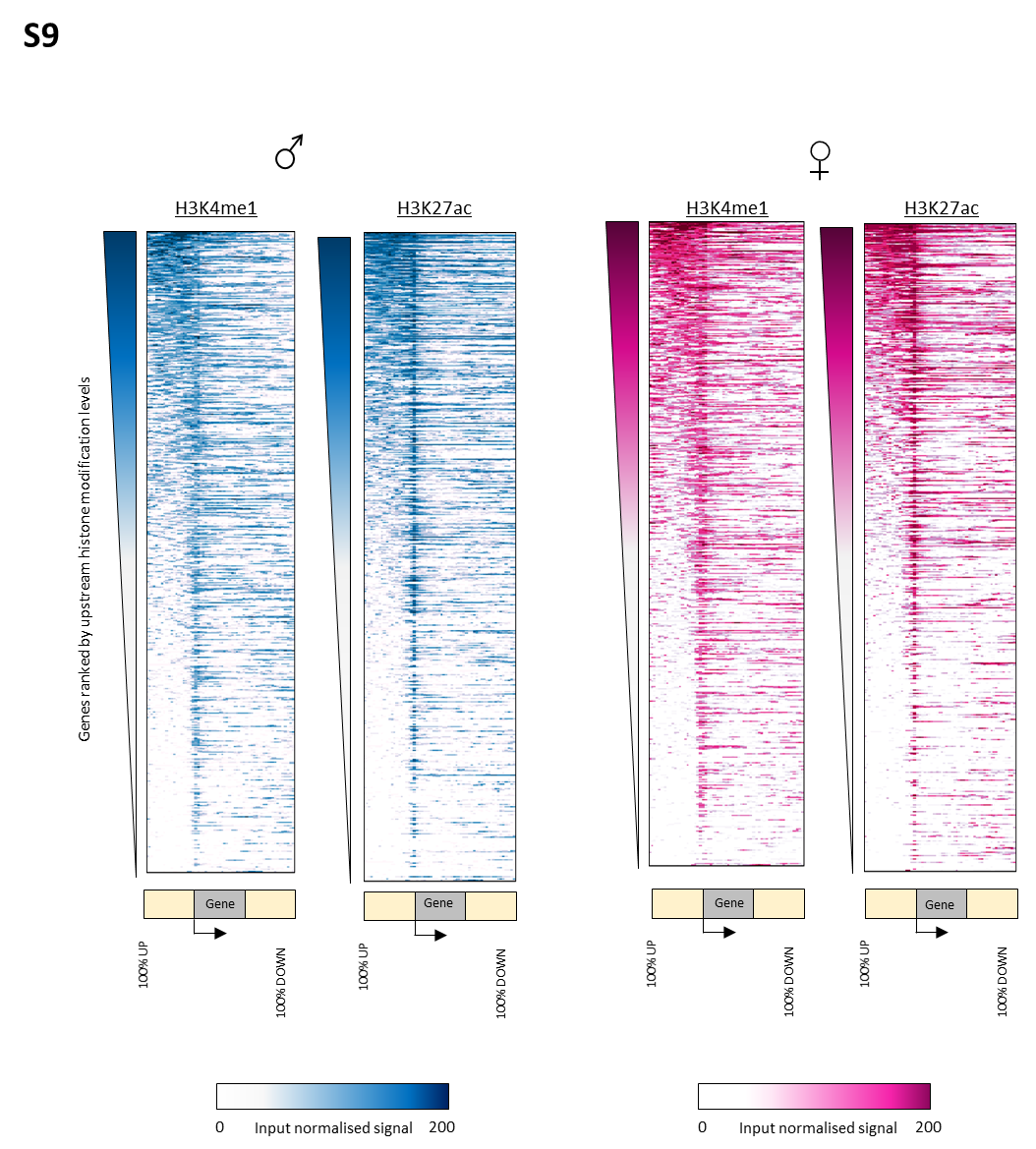** |
| **Figure S9.** Heatmap of average H3K4me1 and H3K27ac patterns across the total gene set of male (blue) and female (pink) Wistar rats. Plots relate to those in figure 4D.Genes are ranked individually in each plot by the total level of histone modification in promoter distal (TSS +2kb to +1kb) regions. |

| **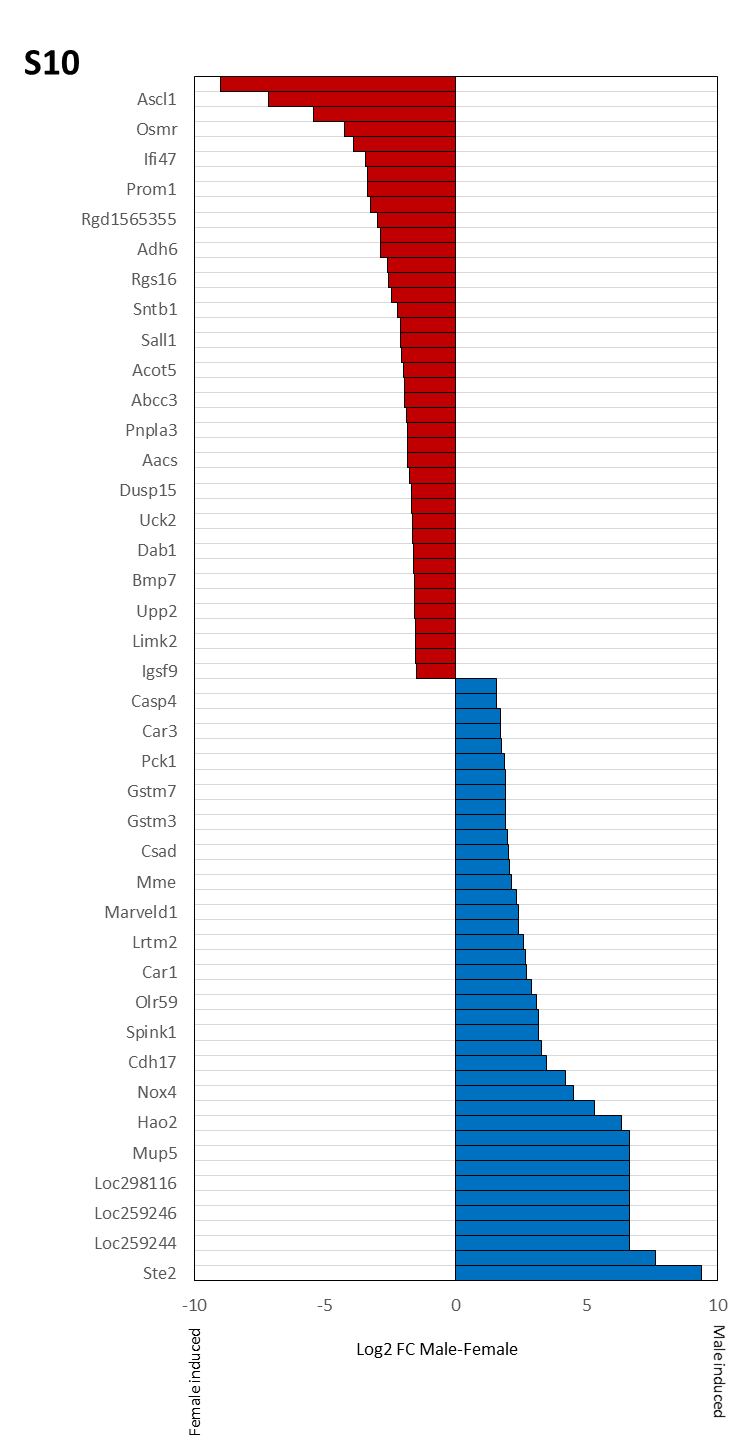** | **Figure S10.** Plot of mean fold change across genes exhibiting gender dependent gene expression between sets of Wistar mouse livers. Red bars represent female specific genes, blue bars represent male specific genes. |
| --- | --- |
